# The anti-crystal engineering principles of imidazolium cations for ionic liquids

**DOI:** 10.1039/d5ce00872g

**Published:** 2025-12-03

**Authors:** Patrick C. Hillesheim, Arsalan Mirjafari

**Affiliations:** a Department of Chemistry, Illinois State University Normal Illinois 61761 USA pchille@ilstu.edu; b Department of Chemistry, State University of New York at Oswego Oswego New York 13126 USA arsalan.mirjafari@oswego.edu

## Abstract

Crystallography provides a powerful framework for identifying, characterizing, and designing new ionic liquids (ILs) with targeted thermal and structural properties. While the design of imidazolium-based ILs has historically relied on empirical modification of alkyl chain length, cation symmetry, and electronic or steric effects, crystallography reveals how these molecular parameters dictate lattice packing, intermolecular interactions, and ultimately melting behavior. Despite extensive study, critical structure–property relationships remain unresolved, including the impact of C4 and C5 methylation, odd-numbered alkyl chains, and conformational polymorphism. From a crystal engineering perspective, the design of low-melting ionic compounds can be viewed as a deliberate inversion of traditional crystal design principles. Rather than promoting long-range order, researchers aim to disrupt specific noncovalent synthons and reduce lattice enthalpy to favor fluidity. This tutorial review unifies these perspectives by examining how crystallography has helped steer structural design to control interactions, torsion angles, molecular descriptors, and hydrogen-bond networks to modulate the behavior of dialkylated imidazolium salts. The discussion highlights how crystallography transforms the empirical art of IL synthesis into a rational, structure-guided design strategy for next-generation materials.

## Abstract 2

Crystal-engineering principles are inverted, revealing how imidazolium cation architecture destabilizes the solid state.

## Graphical abstract

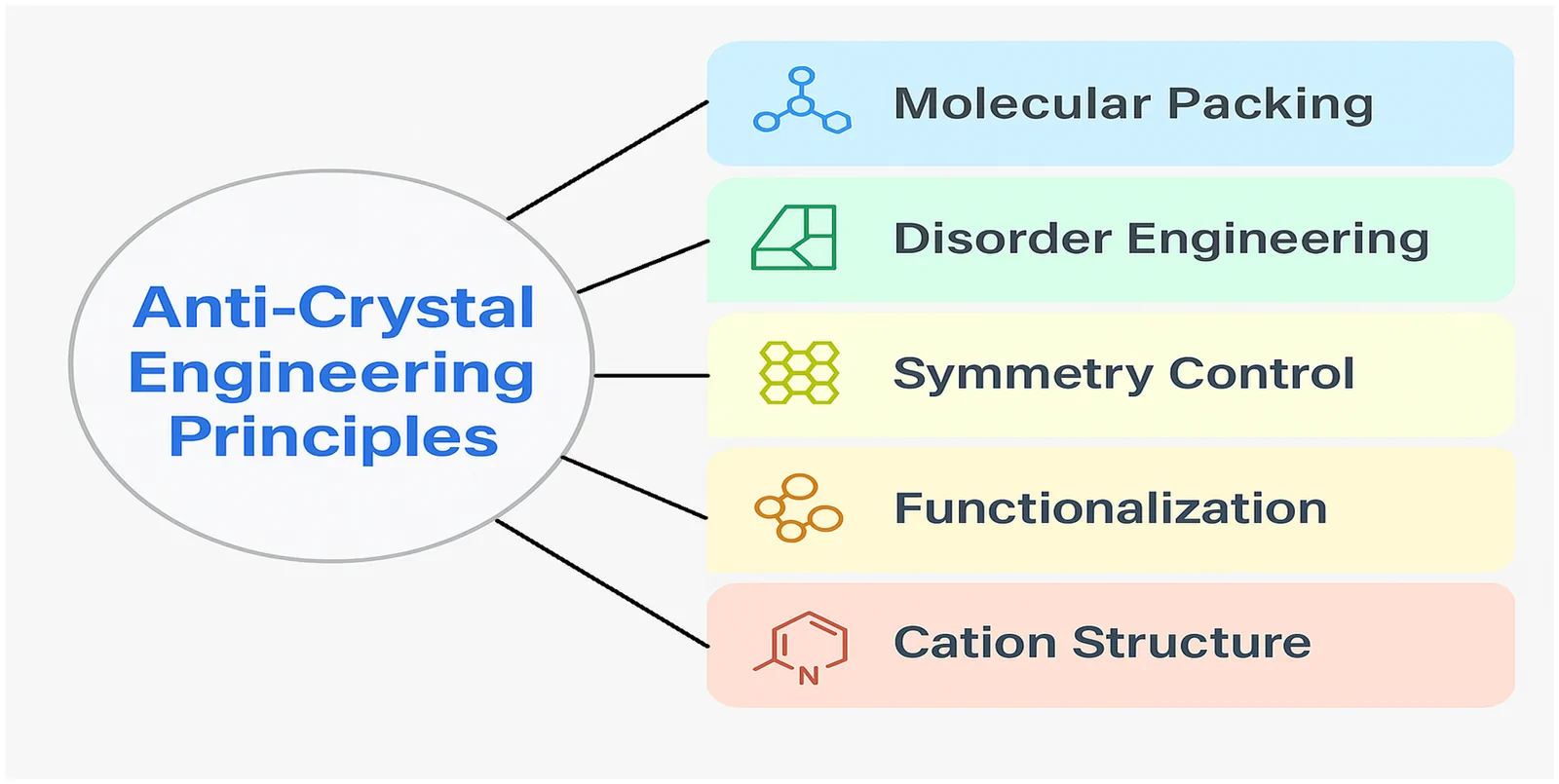

**Key points**

**• Crystal engineering provides a structural framework for understanding ionic liquids.** Crystallographic analysis reveals how molecular geometry, packing motifs, and intermolecular forces govern melting points and phase behavior.

**• Disorder can be deliberately engineered.** Anti-crystal engineering principles, such as cation asymmetry, conformational flexibility, and weak interactions, show how structural frustration suppresses crystallization to yield stable liquids.

**• Symmetry and shape control lattice energy.** Variations in chain length, branching, or methylation alter molecular symmetry and steric balance, tuning lattice enthalpy and entropy to direct solid–liquid transitions.

**• Functionalization of the alkyl chains can lower crystallinity**. Addition of functional groups can disrupt chain packing, lower melting points. However, the functional groups can also introduce new interactions which may, conversely, increase cohesion.

• **The parts of the imidazolium cation**. The imidazolium cation can be viewed as comprising three regions—the charge-rich heterocycle, the symmetry-breaking domain, and the hydrophobic tail—each serving as a modular platform for introducing anti-crystal engineering features.

## 1 Introduction

The development of dialkylated imidazolium salts as low-melting materials in the early 1980s marked the emergence of what are now recognized as ionic liquids (ILs).^1^ These early compounds, typically containing aluminum-based anions such as [AlCl_4_]^−^, emerged from decades of research on aluminum chloride electrolytes.^2^ Consequently, imidazolium ILs can be viewed as the direct descendants of this earlier molten-salt chemistry meaning there is a rich history of IL precursors.

Earlier studies had described related low-melting salts derived from other alkylated heterocycles, including pyridinium species, though terminology varied widely. Historical ambiguities, the definition of “ionic liquid”, limited access to early literature, and the interchangeable use of terms such as “eutectic” or “molten salt”—complicate efforts to assign a single point of origin for ILs.^3–5^ These complications aside, for this present review, we will simply be focusing on the role of crystallography and its impact on the development of dialkylated imidazolium ILs. For those interested, a rigorous analysis of crystallography of ILs was previously reported.^6^

### 1.1 [C_2_mim] and the foundations of imidazolium-based ionic liquids

**Fig. 1:**
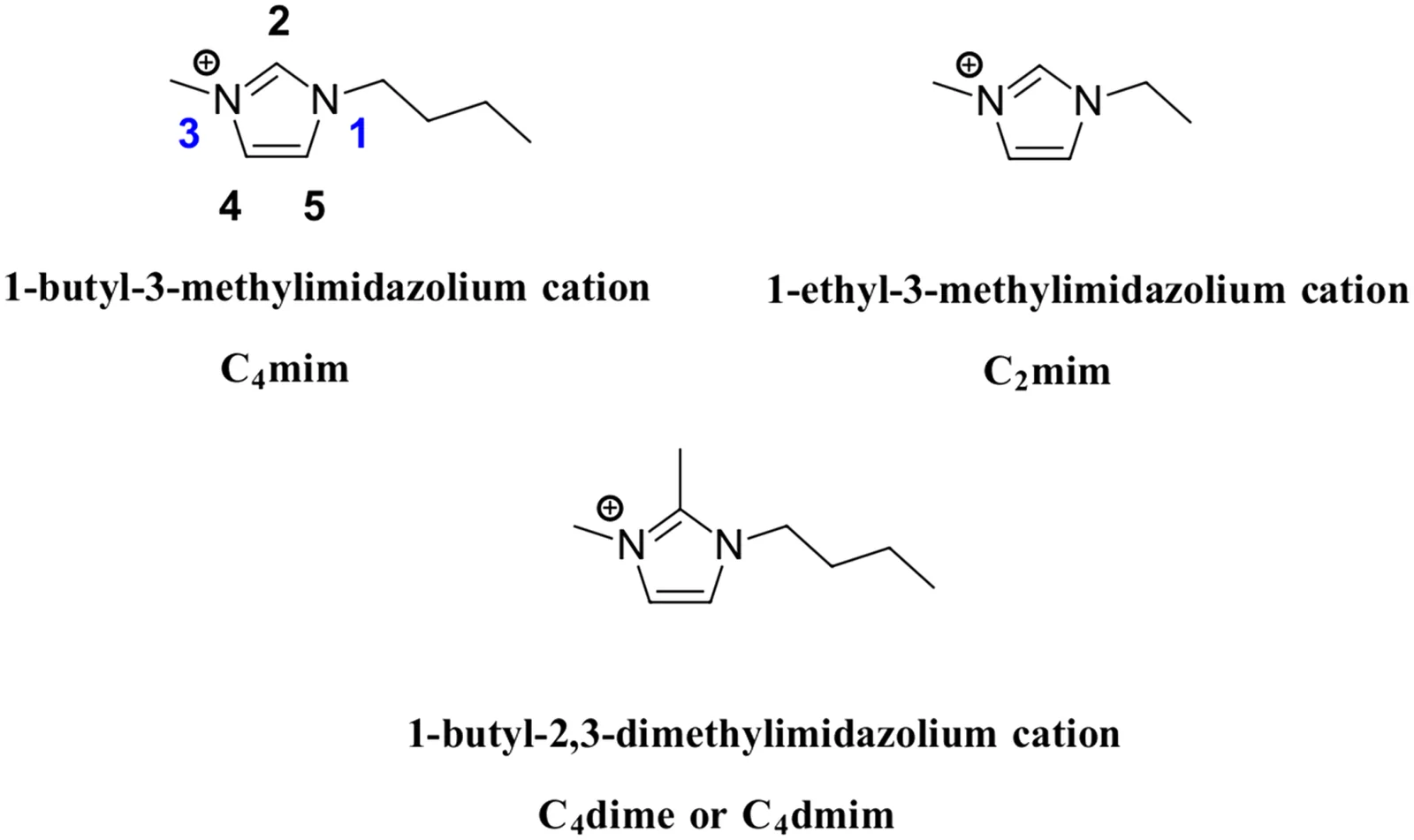
Depiction of prototypical dialkylated imidazolium ionic liquids. Conventional numbering is used for the rings. Naming is shown along with commonly accepted abbreviations.

The earliest dialkylated imidazolium structure in the Cambridge Structural Database was reported in 1986 by Appleby and co-workers, featuring the 1-ethyl-3-methylimidazolium ([C_2_mim], Fig. 1) cation paired with a ruthenium oxide anion.^7^ Although the study focused primarily on synthesis, it used the term “ionic liquid” to describe these salts. Later that year, Seddon and co-workers published the first crystal structure of a prototypical halide IL, [C_2_mim][I], analyzing its hydrogen-bonding network and validating earlier spectroscopic evidence for nonclassical C–H⋯X interactions.^8^ These works mark some of the first crystallographic insights into how hydrogen bonding, cation shape, and packing interactions influence phase behavior in ILs. Simply, these are the groundwork studies for the anti-crystal engineering of ILs.

The genesis of ‘modern’ ILs (*viz.*, air and moisture stable) is the report by John Wilkes and Michael Zaworotko in 1992.^9^ By replacing hydrolysis-prone chloroaluminate anions with charge-disperse, water-stable species through simple salt metathesis,^10^ they generated a family of [C_2_mim]-based salts spanning a broad range of melting points. Their paper reported the crystal structures of [C_2_mim][NO_2_], [C_2_mim][NO_3_], and [C_2_mim]_2_[SO_4_·H_2_O], establishing a structural foundation for correlating molecular design with phase stability. Their crystallographic discussion touched on several principles now central to crystal engineering: the role of cation shape and size in directing packing, the influence of charge dispersion across the imidazolium ring, and the competition between stacking interactions and electrostatic pairing.

In the conclusion of the paper, they write:

“*It is clear from this study that [C*_*2*_*mim] is an ideal candidate for general use in ILs. It is of moderate size…its shape appears to promote cation stacking…and can only engage in thermodynamically weak C–H⋯X hydrogen bonding.*”

We now know that [C_2_mim] is indeed an ideal candidate for ILs, with thousands of published reports using this cation for the successful development of ILs. Indeed, the success of this privileged cation arises, in part, from several important features alluded to in the conclusion of their report: alkyl chain length, cation size (asymmetry), the charge disperse nature of the aromatic imidazolium ring, the weakening of cation interactions, and the ever-important H-bond. Thus, from the early times of ILs, crystallography has played a pivotal role in both guiding the design of ILs as well as providing key details that helped solve some of the earliest mysteries in the field, steering the field to the robust development observed today.

### 1.2 Butylated imidazolium cations

While [C_2_mim] remains central to ionic-liquid research, the butyl analogue, 1-butyl-3-methylimidazolium ([C_4_mim]), occupies an equally iconic role. Together, these cations dominate the IL literature, and, as with [C_2_mim], crystallography has been instrumental in revealing why [C_4_mim] forms liquids so readily, resisting crystallization.

The first reported structure containing [C_4_mim] appeared in 1998, when Prof. Dupont and co-workers used an IL as a catalyst.^11^ Although their focus was synthetic, it marked the debut of this cation in a crystallographic context. Seddon and co-workers followed in 2002, studying ILs as solvents for uranium extraction.^12^ As with the aforementioned study, the focus of this paper was not on the structure of the ILs but rather their application.

The crystallographic story of [C_4_mim]-based ILs truly began in 2003, when the first crystal structures of the halide salts were published. The chloride structure ([C_4_mim][Cl]) was independently reported by two groups,^13,14^ and the bromide structure ([C_4_mim][Br]) by Seddon and Rodgers.^13^ These studies revealed conformational polymorphism in the butyl chain, which can adopt both *gauche* and *anti* conformations that are nearly degenerate in energy. This conformational freedom was proposed to frustrate lattice formation, explaining the common tendency of ILs to supercool rather than crystallize.

Later that same year a report centered on the structure of the [C_4_mim][F] crystal would send ripples across the IL community.^15^ The compound, isolated as a hydrolysis product of [C_4_mim][PF_6_], revealed that the ostensibly “green” [PF_6_]^−^ anion could decompose under ambient conditions, producing fluoride and challenging ILs' reputation as benign solvents.^16^

By the end of that year, nearly the full halide series of [C_4_mim] salts had been characterized, leaving only the elusive iodide. In 2004, Katayanagi and co-workers used scattering and Raman spectroscopy to model the low-melting [C_4_mim][I], extrapolating from the known chloride and bromide structures.^17^ They proposed that *gauche* and *trans* conformations in the butyl chains create hydrophobic regions interspersed with ionic channels. Nakakoshi *et al.* later obtained the first single crystal of [C_4_mim][I], which confirmed these torsional motifs but revealed weaker hydrogen bonds to iodide, explaining its lower stability and melting point.^18^ Their findings helped to validate the previous theories, while also adding a bit of mystery due to the lack of polymorphs observed. The reader should note that the crystal structure of [C_4_mim][I] does not exist in the CSD as of this writing, though is available through the SI in the reference provided.

**Fig. 2:**
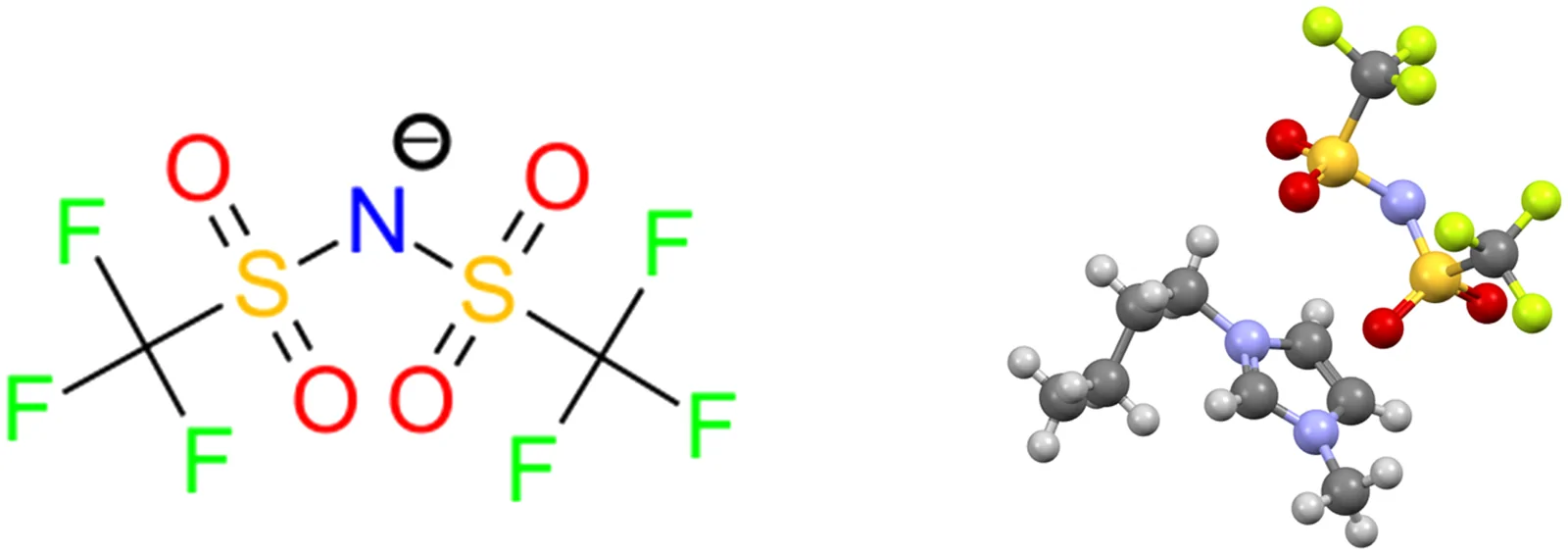
(left) Depiction of the bis(trifluoromethane)sulfonimide anion ([NTf_2_]), one of the most important anions for ionic liquids. (right) The molecular structure of [C_4_mim][NTf_2_] from the crystal structures reported by Paulechka *et al.*^19,20^

When considering the descriptors such as ‘iconic, prototypical, emblematic, benchmark, and problematic (to crystallize)’ ILs, [C_4_mim][NTf_2_] is likely one of the first compounds to spring to mind. Indeed, [C_4_mim][NTf_2_], alone, has over 6300 references in the literature in a recent survey (Fig. 2). Crystallization of [NTf_2_]^−^ salts proved more difficult than for halides, but Paulechka *et al.* eventually reported the first single-crystal structures of [C_2_mim][NTf_2_], [C_4_mim][NTf_2_], and [C_6_mim][NTf_2_].^19,20^ Their study revealed cation conformational polymorphism similar to earlier halides and highlighted *cis*–*trans* isomerism of the [NTf_2_]^−^ anion as a key contributor to low melting points.^21^

These crystallographic milestones underscore a broader principle: some ion combinations crystallize far more readily than others. Halide salts, for example, were structurally characterized long before [C_4_mim][BF_4_], [C_4_mim][PF_6_], or [C_4_mim][NTf_2_], illustrating the importance of anion choice in crystal engineering.^22^ Simply, if one's goal is to crystallize their ILs, anion choice is particularly significant. However, the focus for this current review is on cation design, thus we will point the inclined reader to other sources for those interested in discussion of anion structure.^23^

The story of these [C_4_mim] structures is a prime example of why the crystallographic analysis of ILs remains important. Key structural details, both electronic and molecular, can be gleaned from crystallographic data. It is, of course, inherent to the design of ILs that they are difficult to crystallize. After all, if you are difficult to crystallize, you are more likely to stay as a liquid, which is often the goal in this field (*i.e.*, developing room temperature ionic liquids). In the words of Prof. Mudring:

“*Solidification and especially crystallization of ILs is by no means an easy task and it becomes technically more demanding the lower the melting point of the respective IL is.*”^24^

Despite the challenges inherent with the task of crystallizing ILs, there now exists a sizable library of reported structures.^6^ Despite the many structures, however, questions regarding IL design and properties remain unanswered. Some of these questions are, in the authors' opinions, best approached through thoughtful and rigorous crystallographic studies.

From a crystal-engineering perspective, these observations highlight a central paradox of IL design: the same structural features that make a compound difficult to crystallize also provide the key to understanding its behavior. The frustration of long-range order, conformational flexibility, and weak, diffuse interactions are not obstacles to be overcome but parameters to be quantified and exploited. It is within this delicate balance, between the desire to suppress crystallization and the need to understand it, that the concept of anti-crystal engineering emerges.

### 1.3 The anti-crystal engineering of ionic liquids

It may seem counterintuitive to discuss crystal engineering in the context of materials that are, by virtue of their name, believed to be liquid. After all, crystal engineering traditionally concerns the deliberate assembly of supramolecular architectures through non-covalent interactions (NCIs) to achieve long-range order in the solid state.^25^ However, the solid state lies only marginally to the energetic “left” of the melting point (*i.e.*, solid ⇌ liquid). The equilibrium between the crystalline and liquid phases implies that most substances existing as a liquid likely possess an accessible crystalline form nearby in its thermodynamic landscape. Given this proximity, the study of ILs is inseparable from the study of their crystalline counterparts. In simpler terms, because melting behavior is one of the most frequently discussed properties of ILs, the examination of crystal structures provides essential insight into the molecular interactions, packing motifs, and energetic factors that govern phase stability. In this sense, crystallographic analysis represents not merely a complementary tool but a foundational approach for understanding and rationally designing ILs with tailored melting points.

A landmark example comes from the crystallographic study of [C_2_mim][PF_6_] by Fuller and co-workers,^26^ which foreshadowed many principles now recognized as anti-crystal engineering. Their work highlighted the predominance of electrostatics as the key non-covalent interaction in ILs^27,28^ and demonstrated how the weakly coordinating [PF_6_]^−^ anion can disrupt π-stacking among imidazolium cations. The resulting lattice features unusually long cation–cation separations governed more by steric and electrostatic balance than by classical hydrogen bonding. Fuller's study thus revealed how the same interactions that frustrate long-range order in the liquid also limit structural coherence in the solid, capturing the essence of anti-crystal engineering.

From this and subsequent studies, several general principles emerge. Weakening or eliminating directional hydrogen bonds, such as by C2-methylation, reduces lattice cohesion and forces anion association through weaker C–H donors. Conformational degeneracy introduced by flexible alkyl chains adds multiple local minima to the potential-energy surface, promoting polymorphism and disorder. Cation asymmetry and extended chain length further frustrate efficient packing, generating competing alkyl–alkyl domains that counteract, partially, electrostatic order. Odd–even alternation, polymorphism, and the use of bulky, charge-diffuse cations with weakly coordinating anions (*e.g.*, [PF_6_]^−^, [NTf_2_]^−^) together suppress long-range order through a delicate interplay of steric and electrostatic effects. Collectively, these structural features illustrate how IL design intentionally inverts the logic of traditional crystal engineering—achieving stability not through order, but through controlled molecular disorder.

In the sections that follow, we unpack these anti-crystal engineering concepts in detail, revealing the underlying structural logic that explains why ILs so effectively resist crystallization. As an important reminder, we focus, almost exclusively on imidazolium cation of ILs. Much of the same discussion applies to the anions, but we leave that for a future study.
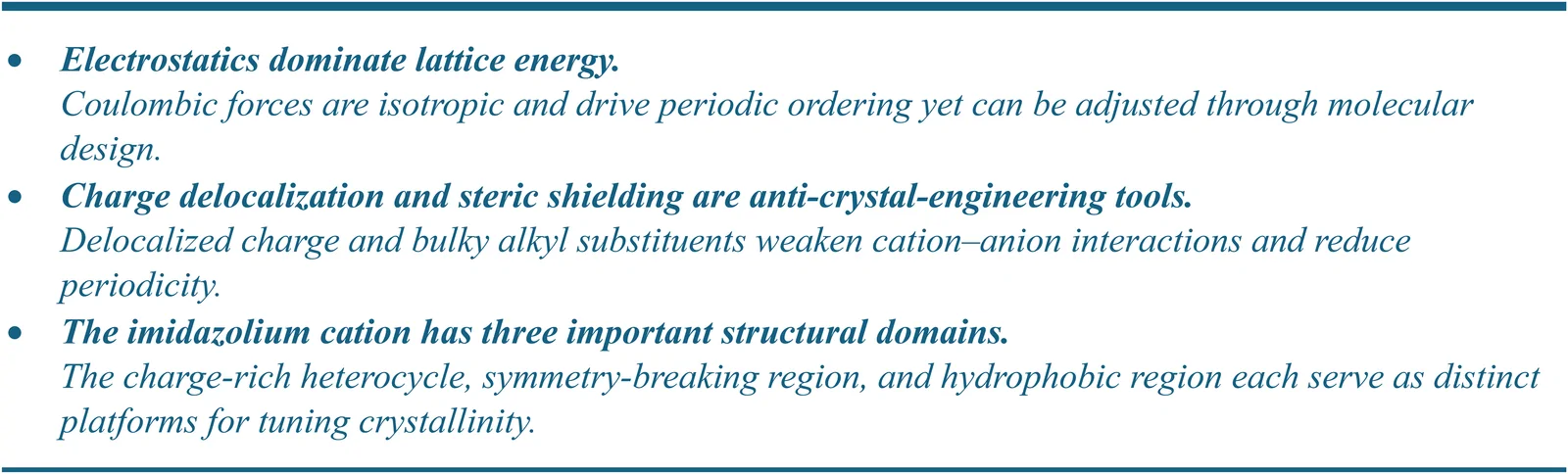


## 2 Anatomy of an imidazolium cation: structural domains and electronic concepts

**Fig. 3:**
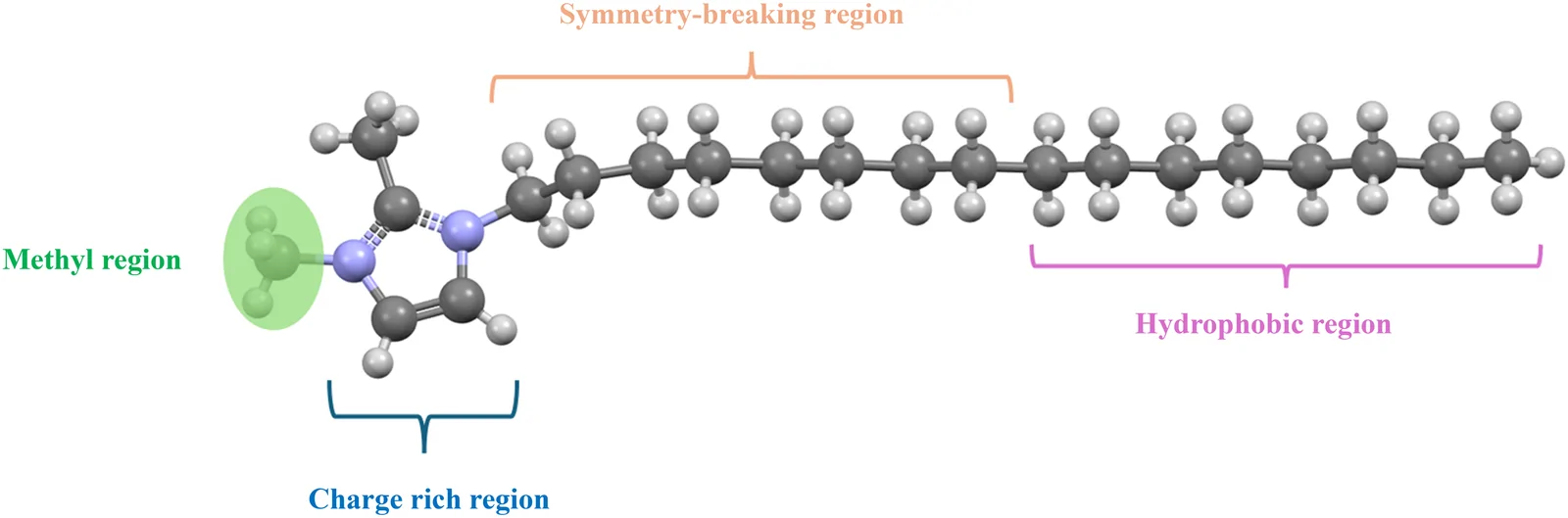
The three regions of the cations for dialkylated imidazolium ionic liquids. The image is inspired by the discussions and figures in ref. 29.

Before examining the anti-crystal-engineering design of di-alkylated imidazolium cations, it is useful to first delineate the distinct structural regions that compose the cation (Fig. 3). Commonly in the literature there are three parts to an imidazolium cation: the charge rich heterocycle, the symmetry breaking region, and the hydrophobic region.^29^ Each of these domains contributes differently to the crystallization landscape of ILs, as steric or electronic modification within any one region alters the balance of interactions that dictate solid-state order.

These three domains provide a convenient framework for discussing the structure of di-alkylated imidazolium cations. For clarity, however, we highlight an additional sub-region—the methyl domain (shown in green)—since much of the discussion in the literature centers on asymmetric cations containing a single methyl substituent. Of course there exist ILs which do not have methyl groups, and could thus have two (or more) symmetry-breaking and hydrophobic regions.^30^ We discuss the hydrophobic and symmetry breaking regions in § 5.2.

From an energetic standpoint, the charge-rich portion of the imidazolium cation exerts the greatest influence on the properties of ILs. This is, after all, the region where the formal positive charge resides, firmly establishing ILs as ionic species. In the context of crystal engineering, this domain defines the electrostatic landscape of the lattice. It is important to remember that coulombic forces dominate the energetic balance of ionic crystals for two reasons: (1) they can be orders of magnitude stronger than other non-covalent interactions,^27,28^ and (2) they are isotropic—acting equally in all directions. From a crystal-engineering perspective, this isotropy promotes structural regularity and periodicity, as oppositely charged ions naturally arrange into repeating ion pairs that minimize lattice energy.

At the same time, however, charge delocalization within the π system of the imidazolium ring and the geometric flexibility of the alkyl groups disrupt periodicity by distributing the electrostatic potential over larger molecular surfaces or with steric blocking, preventing anions for forming close contacts with the cations.^31,32^ In essence, charge delocalization and steric shielding act as anti-crystal engineering strategies, weakening localized charge density and hindering the directional assembly that drives crystallization. Yet these same features provide chemists with powerful structural design tools, allowing deliberate control over crystallization.

For instance, increasing the total cationic charge, such as by incorporating di- or tricationic centers, typically enhances crystallinity.^33^ From a crystal-engineering standpoint, this strengthens the dominant coulombic component of the lattice energy, offsetting the disordering influence of delocalization and asymmetry. Di- and tricationic imidazolium ILs often crystallize more readily than their monocationic analogues due to the combination of stronger electrostatic cohesion, the emergence of directional secondary interactions (*e.g.*, hydrogen bonding and π-stacking), and, in many cases, higher molecular symmetry. Surprisingly, however, even ILs with higher cation charges can exhibit low melting points, with some being below 100 °C,^34^ pointing towards an central concept: while coulombic forces are very important, the molecular architecture of the cations has a significant influence on stabilization of the solid-state.

Zwitterionic compounds present a useful case study in the influence of ion ordering of IL-like species.^35^ In such compounds, the cation and anion are covalently tethered, effectively lowering configurational entropy and promoting long-range order in the solid state.^36^ From a crystal-engineering standpoint, this enforced proximity constrains the ions to adopt a geometry that minimizes charge separation and maximizes electrostatic complementarity, thereby stabilizing a well-defined lattice.^37^

By contrast, in conventional ILs the ions are discrete and mobile, enabling multiple energetically similar cation–anion arrangements to coexist. This dynamic degeneracy, where numerous local minima exist on the potential energy surface, introduces a form of structural frustration that resists the formation of a single dominant packing motif. Consequently, while zwitterions can exemplify the ordered extreme of coulombic organization, ILs occupy the opposite end of the spectrum: a landscape of competing weak interactions and entropic freedom that embodies the principles of anti-crystal engineering (see § 4.2, Polymorphism).

While our discussion is on the interplay between molecular and crystal structure, there is, of course, the electronic structure of the imidazolium cation to consider. Of particular importance is the distribution of π electrons within the aromatic portion of the heterocycle (*e.g.*, charge delocalization). Quite often imidazolium cations are drawn with a completely delocalized positive charge within the ring. Certainly this is a ‘valid’ representation, though the reality of the electronic structure is more complex.^38^

**Fig. 4:**
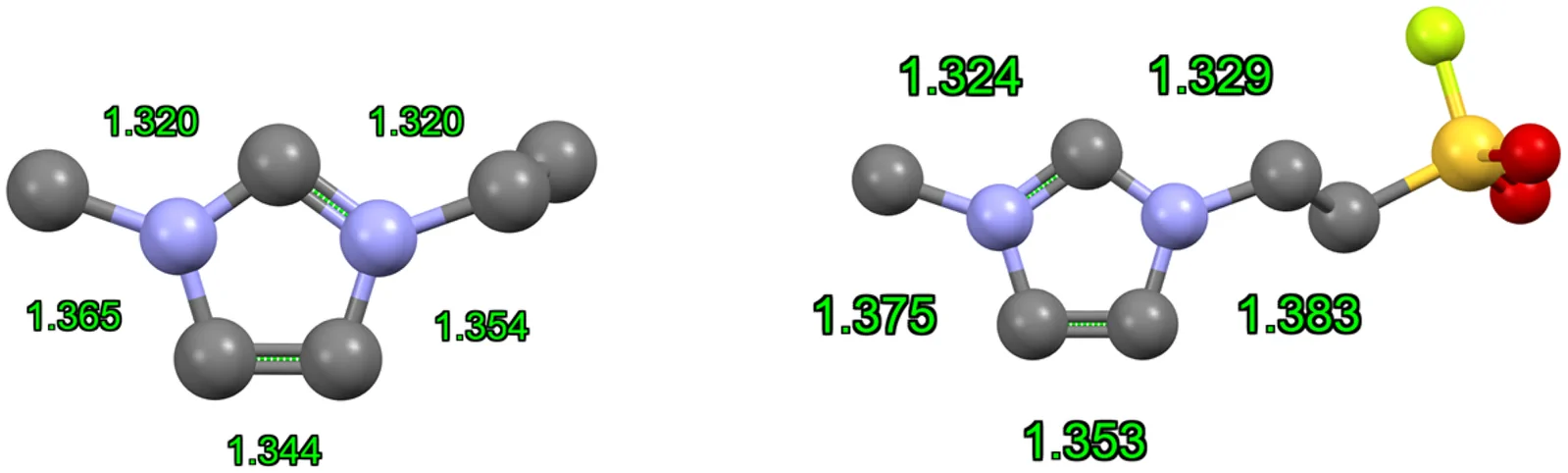
Two examples of dialkylated cations with bond distances shown in green. The bond distances point towards the charge delocalized N–C–N region of the imidazolium ring. (left) The [C_2_mim] cation is from ref. 9 (right) a [C_2_mim] derivative from ref. 40 as a contemporary example.

According to the computational study by Hunt *et al.*, a more accurate electronic representation would entail a 3-center, 4-electron bond spanning the N1–C2–N3 portion of the ring, with a distinct π bond between the C4–C5 carbons.^39^ From a crystallographic standpoint, the theoretical model is borne out by systematic variations in bond distances within the ring: a fully delocalized π system would produce uniform N–C and C–C lengths, whereas experimentally determined structures consistently reveal alternating shorter and longer bonds. This pattern, evident in both early IL crystal structures^9^ and more recent higher resolution structures,^40^ supports partial delocalization of the positive charge along the N–C–N moiety and a distinct C4=C5 π bond (Fig. 4).

In summary, the dialkyl-imidazolium cation presents an intricate balance of charge delocalization, molecular symmetry, and structural anisotropy that collectively define its crystal-engineering behavior. The π-electron topology of the ring dictates where and how anions can approach, while delocalization and steric modulation within the alkyl domains tune the strength and directionality of these contacts. From this perspective, the imidazolium scaffold is not merely a passive charge carrier but a finely adjustable structural platform in which small perturbations—such as substituent type, position, or symmetry—can alter packing preferences and melting behavior. The next logical question, then, is how targeted chemical modifications such as methylation or addition of functional groups, reshape these delicate balances. In the following sections, we examine methylation as a prototypical example of anti-crystal-engineering design, where deliberate disruption of strong intermolecular synthons allows for control over the crystallinity of ILs.
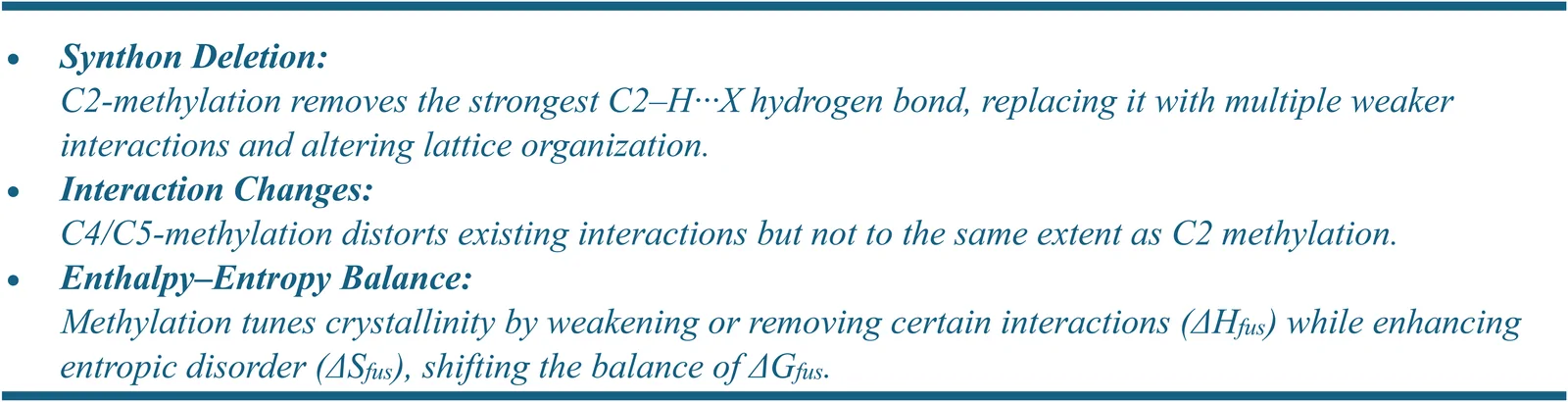


## 3 To me or not to me: comparative insights into methylated cations

### 3.1 C2 methylation

Methylation offers a particularly instructive lens through which to view anti-crystal engineering in ILs, as even the smallest substitution on the imidazolium ring profoundly alters supramolecular organization, packing motifs, and phase behavior. Among the simplest and most instructive examples of anti-crystal engineering in imidazolium systems is substitution at the C2 position of the heterocycle. For example, methylation at the C2 position of [C_2_mim][NTf_2_] increases the melting point from −17 °C to 25 °C.^41^ Similarly, C2 methylation of [C_4_mim][PF_6_] results in a change of 11 °C to 40 °C.^42^ Notably, this was not the expected outcome when these C2 methylated ILs were first developed.^43^ The same increase in melting points (*i.e.*, increased crystallinity) upon methylation is observed with changing alkyl groups or anions. At first glance, this trend appears paradoxical: replacing a hydrogen with a methyl group might be expected to weaken cohesive forces. Crystallographically, however, the effect reflects a classic example of synthon disruption: the removal of a strong, directional H⋯X hydrogen bond that normally links cation and anion into extended motifs.

As briefly mentioned in the introduction, hydrogen bonding in ILs has and continues to be an important focus of study within ILs.^44^ With respect to the imidazolium heterocycle, there exist three aromatic hydrogens at the C2, C4, and C5 positions. All three of these hydrogens participate, predominantly, in H⋯anion interactions. Of these three aromatic hydrogens, the ‘central’ C2 hydrogen has received the most attention due to its acidity, exhibiting a p*K*_a_ ranging from *ca.* 16 to 24 depending on solvent and the nature of the pendant alkyl chains.^45^ Thus, while imidazolium ILs are the most studied heterocyclic cations, the ‘acidic’ C2–H limits application of imidazolium ILs under strongly basic conditions in addition to decreasing thermal stability *via* thermally induced deprotonation mechanisms.^46,47^ On the other hand, the acidity of the C2–H has been leveraged extensively in the formation of carbenes.^48^

The earliest report of a 1,2-dimethylimidazolium cation is from 1925 wherein the synthesis and characterization of a series of protonated salts was discussed, mirroring other early reports of ILs formed from neutralization reactions (*e.g.*, ethanolamine and nitric acid, see Conclusion).^49^ With the active discussion of hydrogen bonding in Mim-based ILs, researchers were quick to add a methyl group to the central C2 position, ushering in the dimethylimidazolium (Dime or Dmim) subclass of ILs.

The first reported crystal, in the CSD, of a Dime-based IL is from Profs. Kenneth Seddon and Thomas Welton's groups in 1990.^50^ Within their report, the authors point towards the presence of short contacts (less than the sum van der Waals radii) between the anions and the aromatic C4 and C5 hydrogens on the imidazolium cation. They noted that these distances were longer than with the C2–H of an imidazolium cation and thus as being weaker interactions.

**Fig. 5:**
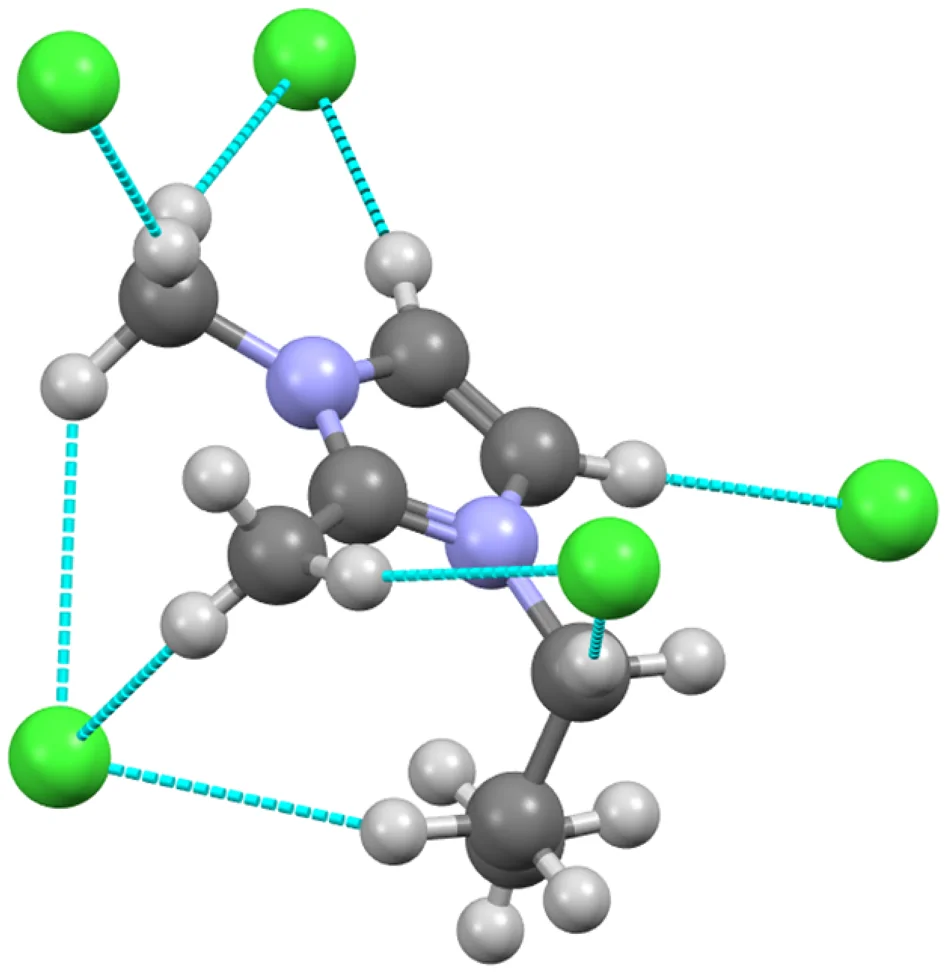
The cation of the crystal structure of [C_3_Dime][Cl]^51^ shown surrounded by multiple interacting chloride anions. Interactions between the cation hydrogens and the chloride anions are shown as the dashed blue lines.

A follow up study on Dime-based ILs was reported in 1993 by Scordilis-Kelley *et al.*^51^ This paper is relevant for two principal considerations in this setting. First, the manuscript clarifies and expands upon the earlier report by Seddon and Welton of hydrogen bonding in [Dime] structures by discussing the interactions with all the methyl hydrogens, a key interaction in all imidazolium ILs (Fig. 5). The authors point out that the anions do indeed form H interactions with these CH_3_ moieties, albeit at longer distances, as expected. Secondly, the paper is unique in that it is one of the very few reports of an IL crystal with an odd numbered alkyl chain (propyl). We later elaborate on the odd–even effect (see § 4.1).

In both manuscripts, it is noted that the melting points of these Dime-ILs are higher than their Mim congeners. So, the question arises as to why do Dime-based ILs typically have higher melting points if they display weaker hydrogen interactions?

H-bonds are directional, with their optimal X–H⋯A angle (≈150–180°) and H⋯A separation lying in a somewhat narrow range.^52^ This contrasts with largely distance-controlled interactions (*e.g.*, London dispersion forces) whose angular dependence is usually weaker.^53^ Consequently, the C2–H of an imidazolium cation can engage in moderate-to-strong hydrogen bonding depending on the anion's basicity.^54^ Such interactions contribute to the crystal lattice enthalpy and influence the melting point, although the net effect also reflects packing entropy and other non-covalent forces.

When the central carbon is methylated, the anions shift from forming the central C2–H hydrogen bond to forming other interactions. For example, it is observed that C2 methylation results in shorter anion interactions with the C4–H and C5–H aromatic hydrogens. Curiously, this is dependent on the identity of the anion, with halides showing this trend but PF_6_ showing longer interactions.^55^ As the anions shift away from the C2–H to other positions to maximize interactions, this also causes conformational changes in the cation, with different alkyl chain orientations being observed concomitant with C2 methylation. The different alkyl chain conformations, thus, form new interactions leading to the formation of hydrophilic and hydrophobic domains in the solids.^56^

To simplify, methylation removes the formation of the ‘strongest’ H-bond in an imidazolium IL. The anions shift to allow other interactions, which in turn can cause conformational changes in the cations leading to the formation of either new interactions or increasing the percentage of other interactions to compensate (*i.e.*, π–π stacking, C–H⋯π, H⋯H).

In the language of crystal engineering, C2-methylation exemplifies synthon disruption: the intentional removal of a key directional interaction to modulate packing efficiency and phase stability. By preventing the formation of certain stronger hydrogen-bonds and promoting multiple weak interactions, new synthons form (*e.g.*, π-stacking, weaker H⋯anion, alkyl⋯π). Energetically, we do lose a strong hydrogen bond, but the diverse structure of ILs allows for other interactions to form, compensating for a portion of the lattice energy.

A final yet very important fact to keep in mind, is that we are most commonly discussing, or evaluating, atom-to-atom distances for these interactions, thus dealing with enthalpic structural arguments (Δ*H*_fus_). Entropy (Δ*S*_fus_), however, is a significant contributing factor to the changes in melting points for ILs, particularly those with shorter alkyl chains.^57^ The fundamental relationship Δ*G*_fus_ = Δ*H*_fus_ – *T*_m_Δ*S*_fus_ demonstrates that both enthalpic and entropic contributions determine the overall thermodynamic behavior. Consequently, structural interpretations based solely on intermolecular distances provide an incomplete picture of melting point trends (or most other properties), as they cannot account for the entropic effects that may either reinforce or oppose the enthalpic contributions observed through crystallographic analysis. Crystallography can help rationalize the enthalpic component, how interactions strengthen or weaken, whereas the entropic term reflects the conformational degeneracy that often escapes direct structural observation.

### 3.2 C4 and C5 methylation

The case for methylation at the C4 and/or C5 positions is quite distinct when contrasted with the C2 methylation. There are 2836 structures for any 1,3-dialkylated imidazoliums having C2, C4, and C5 hydrogens and 365 reported with a Dime-cation (C2 methyl, C4/5 H). A search for a mim-based cations with any alkyl group in the C4 or C5 position reveals 9 reported structures. These 9 structures come from five total papers, three of which involve the discussion of ILs and their properties, but do not contain detailed crystallographic analyses relevant to changes interactions due to methylation.^58–60^ The remaining papers are dealing with matters outside the field of ILs.^61,62^ Thus, crystal structures of these C4/C5 alkylated ILs are exceedingly rare.

To briefly summarize the structural impacts based on available thermophysical and spectroscopic data, methylation at the C4 or C5 position was shown to increase melting point, but not to the extent of that observed with C2-methylation.^43,63^ To note, however, is that the papers specifically show that methylation at these positions change the interactions between the cations and anion, which in turn affects both the enthalpic and entropic components of the ionic liquids. However, there are no supporting crystal structures with analysis to dig deeper into these findings, yet.

Dimethylation, that is methyl groups at both the C4 and C5 positions leads to higher viscosities.^64^ Thus we can infer that methylation at the C4 or C5 position does have a similar structural impact as with C2 methylation, but not to the same extent. Structurally, a methyl group at the C4 or C5 does not remove the strongest hydrogen-bonding proton, so their effects on melting/solidification are more related to packing, perhaps having a larger influence from entropic factors rather than enthalpic.

### 3.3 Summary

From a crystal-engineering standpoint, these substitutions exemplify disruption of interaction synthons by weakening or geometrical misalignment of existing interaction sites rather than their removal. The resulting packing frustration likely enhances configurational entropy in the solid state and modifies the balance between electrostatic and dispersive interactions. In the broader context of anti-crystal engineering, these observations illustrate how even small peripheral modifications can fine-tune the degree of structural frustration within an IL. Whereas C2-methylation operates through synthon disruption, C4 and C5 substitutions modulate crystal stability by distorting interaction geometry and increasing conformational competition, hence the increase in viscosities and densities which is related to changes in intermolecular forces.^65^ Simply, while the C4 and C5 hydrogens can, and do, form interactions with the anions, methylation shifts these interactions. Together, these methylation patterns reveal that control over crystallization in ILs arises not from a single disruptive feature, but from a continuum of structural strategies that balance enthalpic weakening with entropic gain.

Methylation represents one of the earliest and most effective strategies for tuning the supramolecular landscape of ILs. Each substitution targets the hierarchy of intermolecular interactions—removing, distorting, or competing with established synthons to control how efficiently molecules can pack. Whether by deleting the key C2–H⋯X hydrogen bond, introducing steric bulk into the ring plane, or modulating alkyl-chain conformation, methylation provides a modular and reproducible means of manipulating the balance between order and disorder. In this sense, methylation is not merely a structural modification but a foundational crystal-engineering tool for allowing for control of the degree of crystallinity in ILs.
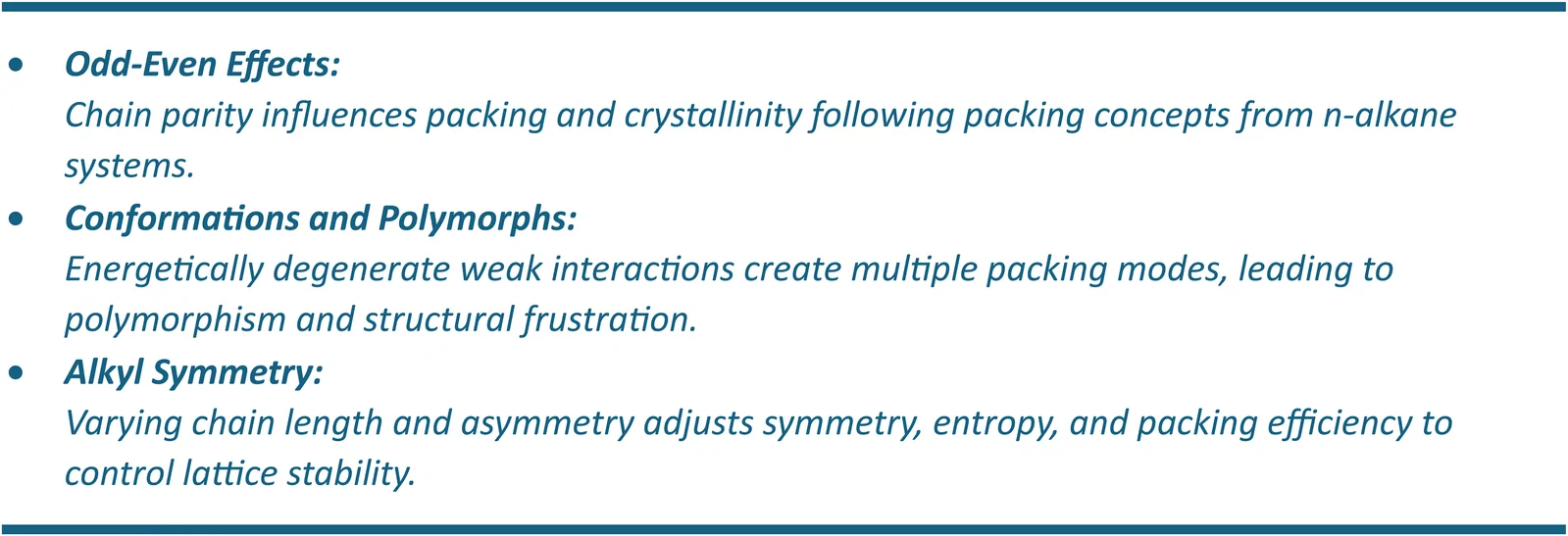


## 4 The alkyl chain of command

The alkyl chains are the second major point of crystallographic engineering for ILs. Indeed, one of the more common studies within the realm of ILs is to examine the impact of increasing alkyl chain length on the specific property the authors are interested in, most commonly melting points.^3^ However, if you pick any of the myriad studies of ILs with increasing alkyl chain length, one will notice that the chosen alkyl chains are nearly always examining even numbered (*e.g.*, ethyl, butyl, hexyl and so on). The reason for this could be attributed to ‘convention’ or perhaps to the human tendency to prefer even numbers to odd.^66^ However, a deeper explanation may lie in the classical odd–even effect, a well-established crystallographic phenomenon in *n*-alkyl systems where chain parity governs packing geometry and, consequently, material properties.^67^

### 4.1 The odd–even effect

With respect to fundamentals of crystal engineering, the odd–even effect is one of the more well-known crystallographic phenomena, finding its roots in the structural ordering of alkyl chains in the solid state.^68^ The odd–even effect posits that the thermophysical properties of alkanes, or alkane-containing molecules (*i.e.*, fatty acids), do not increase in a linear manner with increasing carbon count. Rather, a zig-zag (or sawtooth) pattern emerges wherein sequentially larger odd and even numbered chains show alternating increases and decreases arising due to conformational preferences and packing efficiency.^69^ Simply, the odd–even effect refers to the alternation of physical properties depending on whether the number of methylene groups in the alkyl chain is odd or even—that is, the chain parity of the substituent.^70^

This effect is well-known to impact solid-state properties (*e.g.*, melting points or densities),^71^ but has been shown to manifest in some molten or liquid properties as well.^67^ It is tempting to speculate that early IL researchers recognized this relationship implicitly, as even-numbered chains (*e.g.*, ethyl, butyl, hexyl) quickly became the “standard” series in IL design—despite the fact that even-numbered *n*-alkanes possess higher melting points than their odd-numbered counterparts.^72^ While the odd–even effect is certainly a component of the properties of some ILs, the inherent complexity of ILs tends to defy ‘simple’ models to explain melting points, though there are some notable successes.^73^

While the odd–even effect is not explicitly invoked within their manuscript, Endo *et al.* completed a systematic evaluation of the crystal structures of [C_*n*_mim][PF_6_] (*n* = 1–4).^74^ Two key observations arise. First, polymorphism is prevalent: both [C_4_Dime][PF_6_] and [C_4_mim][PF_6_] display multiple crystalline forms, consistent with prior studies such as the comprehensive work of Hardacre and co-workers.^75^ Polymorphism complicates the interpretation of melting points and can obscure the subtle alternation expected from the odd–even effect.^76,77^ However, polymorphism is a key crystallographic in ILs (see § 4.2, Polymorphism).

Second, while the melting points, specifically, for this [C_*n*_mim][PF_6_] series of ILs does not exhibit the alternating pattern of the odd–even effect, the thermodynamic properties of the compounds do. Examining either the Δ*H*_fus_ or Δ*S*_fus_ of the compounds shows the alternating pattern wherein the odd numbered species have lower values than the even. For example, Δ*H*_fus_ for the series is 17.3 kJ mol^−1^, 19.0 kJ mol^−1^, 16.8 kJ mol^−1^ for the series of [C_1_–C_3_mim][PF_6_], displaying a classical example of an odd–even effect. However, the polymorphs and [C_4_mim][PF_6_] do complicate this pattern, breaking the expected trend in this case.

In the context of crystal engineering, the odd–even effects of the alkyl chains, particularly their influence on packing motifs and long-range ordering, often manifest in subtle yet consequential ways. In certain IL families, direct evidence of parity-driven alternation appears in bulk properties such as melting point and conductivity,^78^ but sometimes, the underlying energetic principles governing phase transitions reveal the effect more clearly. Reports of preferential alkyl-chain conformations in ILs with odd-numbered chains further support this idea, suggesting that chain parity influences both solid-state organization and dynamic behavior.^79,80^ Although a comprehensive meta-analysis of these trends remains lacking, the consistent observation of parity-dependent conformations points toward a broader structural role of the alkyl chain as a key anti-crystal engineering variable—one that not only tunes intermolecular interactions but also promotes the conformational degeneracy central to polymorphism in ILs.

### 4.2 Polymorphism

From a crystal-engineering perspective, polymorphism in ILs arises naturally from the assorted weak interactions that coexist in the solid state. The highly symmetric [PF_6_]^−^ anion, for example, can form several energetically comparable H⋯F contacts with both aromatic and aliphatic regions of the cation. As a result, the anion can adopt multiple orientations around the cation, while the cation itself can assume various rotational conformations without significant energetic penalty. These multiple, near-degenerate interaction patterns give rise to alternative packing arrangements with comparable lattice energies.^81^

Within the framework of crystal engineering, the mechanism of polymorph formation in ILs can therefore be rationalized as a direct manifestation of interaction degeneracy and packing frustration—the design logic that reinforces the broader concepts of anti-crystal engineering.^82^ IL polymorphism reflects synthon competition among weak C–H⋯F and H⋯X contacts and geometric biasing of interaction motifs, where conformational and rotational freedom enable multiple packing solutions of nearly equivalent enthalpy.^83^ This polymorphic flexibility highlights how the very features that lower lattice order—delocalized charge, weaker interactions, and conformational freedom—also provide ILs with a remarkable capacity for structural diversity and tunable crystallinity.

### 4.3 Alkyl conformations

An important finding in the computational study by Bernardino *et al.*,^84^—and one well supported by crystallographic observations—is that the dihedral angle at the point where an alkyl chain attaches to the imidazolium ring has a significant influence on the melting points of ILs. This result establishes a direct link between melting behavior and conformational flexibility within the alkyl chains, offering a structural rationale for tuning thermal properties through molecular design.

**Fig. 6:**
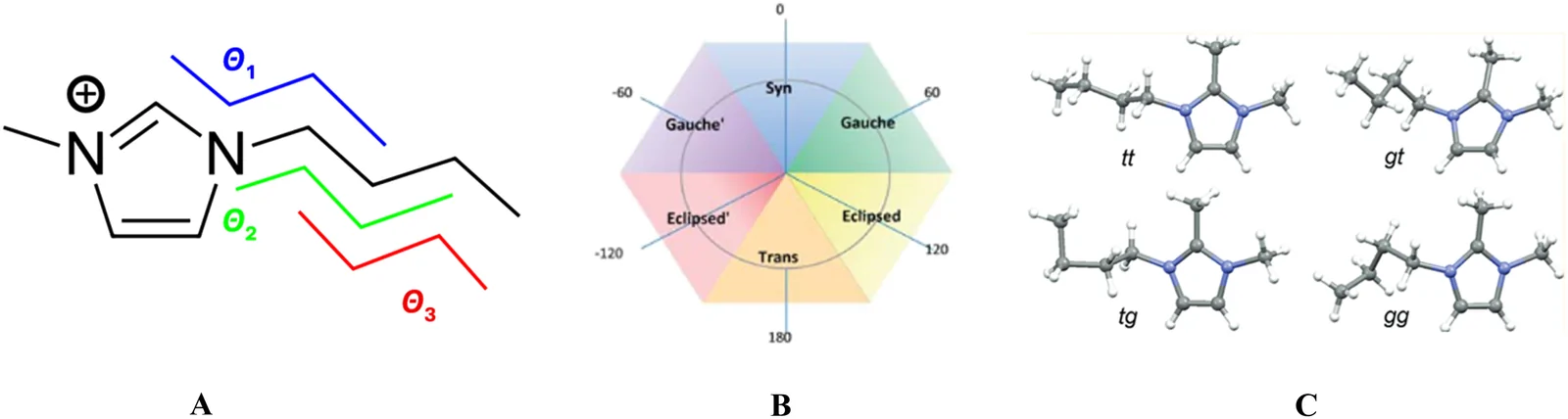
(A) *θ*_1_, *θ*_2_, *θ*_3_ represent key torsion angles for dialkylated ILs. (B) The commonly accepted naming system for these torsion angles. Reproduced from ref. 75 with permission from the Royal Society of Chemistry, copyright 2013. (C) Examples of the [C_4_Dime] cation with different torsion angles. Reproduced from ref. 86 with permission from the American Chemical Society, copyright 2012.

**Fig. 7:**
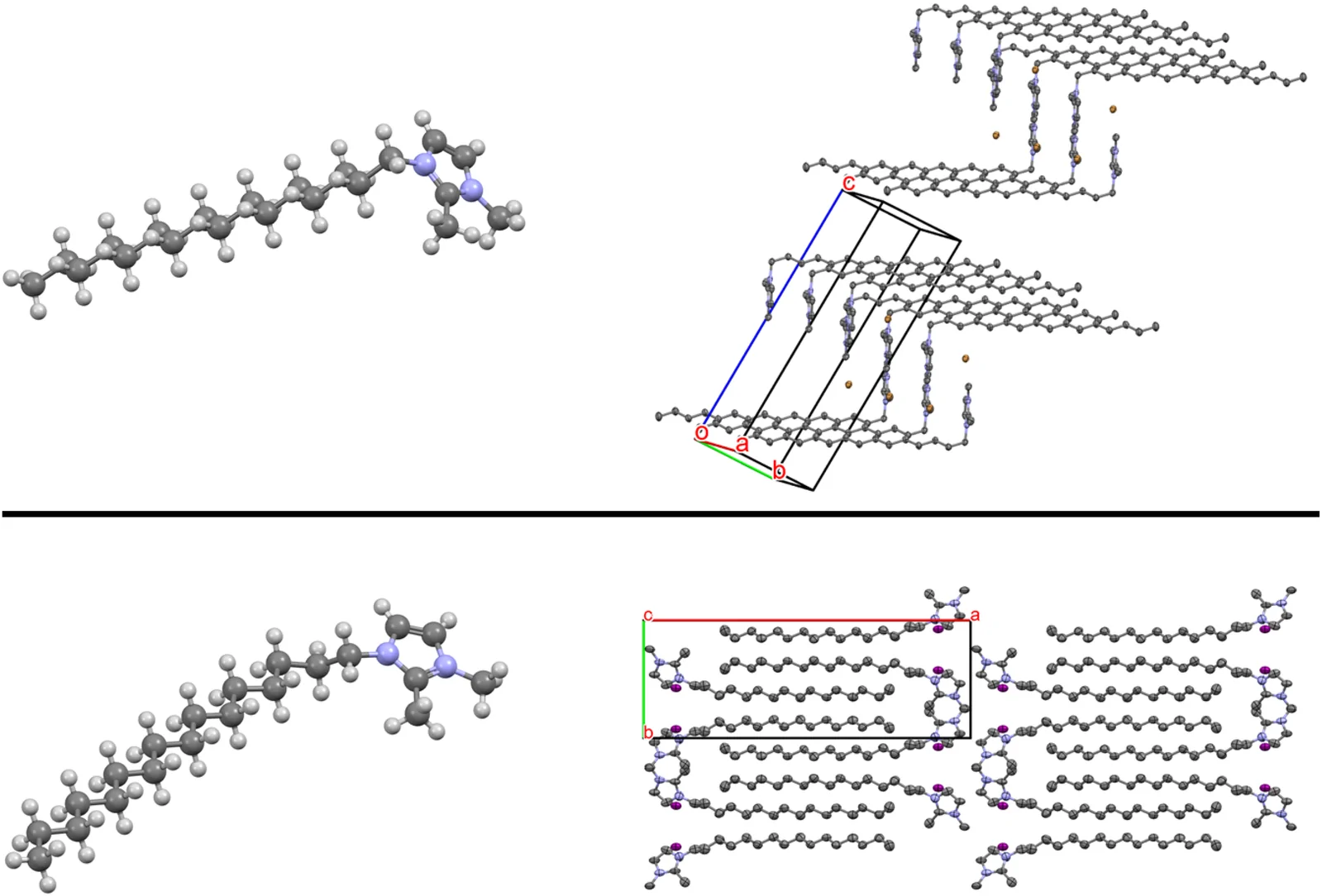
(top) The [C_16_Dime][Br] structure with a packing diagram. (bottom) [C_16_Dime][I] showing a distinct torsion angle in the alkyl chain.^85^ A packing diagram is shown viewed down the crystallographic *c* axis. Changes in the torsion angles affect long range packing (interdigitation *vs.* end-to-end), yet maximizing the alkyl–alkyl interactions is a strong driving force. Anions and hydrogen atoms omitted for clarity in the packing diagrams.

Three dihedral angles deserve particular attention (Fig. 6). Multiple conformations of these angles have been observed in crystal structures of ILs such as [C_4_mim][PF_6_]^75^ and [C_4_mim][Cl],^13^ as examples. Saouane *et al.*^75^ surveyed all available [C_4_mim]^+^ structures, available in 2013, and found that about 30% adopt the TT conformation, while G′T and TG each account for roughly 18%. Fig. 6C illustrates an example of some conformers and the corresponding torsion angles. The effect of these conformations on alkyl packing becomes increasingly pronounced with chain length.^79^ Looking at the structures of [C_16_Dime][Br] and [C_16_Dime][I] typifies the impact of different alkyl torsion angles on the long-range ordering and subsequent properties (Fig. 7).^85^

Conformational freedom within the alkyl chains is important, regardless of chain length. Restricting torsional motion increases crystallinity and raises the melting point, a relationship clearly seen in the C2-methylated cations discussed earlier (§ 3.1). In essence, methylation limits rotational degrees of freedom and reduces configurational entropy, stabilizing the solid phase. Within the framework of crystal engineering, this represents an entropic contribution to lattice stability: reduced conformational diversity enables more efficient packing and a higher degree of order, in essence increasing symmetry (§ 4.3). A study by Laus *et al.* examined a series of Dime-based IL crystals, examining the different torsion angles present within the cation.^86^ They conclude that while the Dime cation does possess a range of energetically accessible conformations such as TT and G′T, certain conformations wherein the alkyl chain is coplanar with the central C2–CH_3_ may not be observable in the solid state due to higher energy.

Further crystallographic analysis supports this restriction of torsional access. A recent study of [C_2_Dime]-based ILs revealed that the most common *θ*_1_ dihedral angles lie in the ranges of 80–85° and 145–150°, corresponding approximately to the *gauche* and *eclipsed* conformers observed in longer-chain analogues.^87^ Co-planar arrangements (175–180°), where the alkyl chain lies in the plane of the imidazolium ring group directed toward the aromatic C5–H, are rarely observed crystallographically, though some examples exist. These combined computational and crystallographic results reinforce the principle that restricting torsional freedom limits conformational diversity, thereby enhancing packing efficiency, lattice stability, and ultimately, the melting point.

### 4.4 A tale of two tails: cation asymmetry

**Fig. 8:**
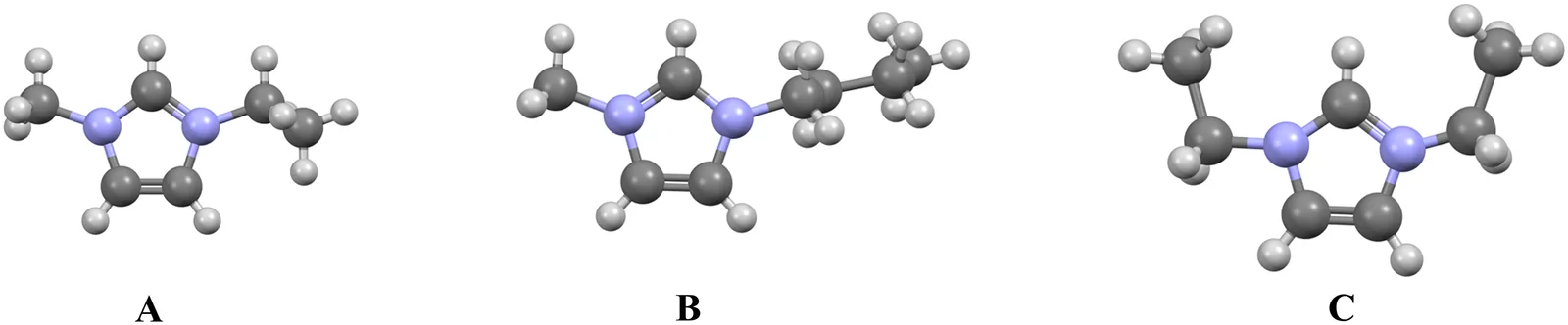
A representation of the cations of [C_2_mim]^90^ (A) and [C_4_mim]^13^ (B). The [C_2_mim] cation is ‘more’ symmetric than the [C_4_mim], but both are considered less symmetric than the diethyl or [C_2_C_2_im] cation (C).^91^

A defining structural feature of low-melting imidazolium ILs is an asymmetric cation imparted by different alkyl chain lengths. A simple inspection would suggest that both [C_2_mim] and [C_4_mim] are less symmetric than an imidazolium with two ethyl chains on the nitrogens (Fig. 8). But how much “more” symmetric is [C_2_mim] than [C_4_mim]? While we can intuit these differences in symmetry, quantifying them is more challenging, though methods do exist (*e.g.*, continuous symmetry measurements).^88,89^

In the late 19th century, Prof. Thomas Carnelley posited an empirical rule that, broadly, higher symmetry corresponds to higher melting points; this has since come to be known as Carnelley's Rule.^92,93^ Although Carnelley's Rule has been widely applied in other fields, few references invoke it explicitly when discussing ILs despite asymmetry being a fundamental design principle.^94–97^ One notable exception is a 2000 publication by R. J. C. Brown and R. F. C. Brown, wherein they specifically discuss “recently developed low-melting ionic materials”, referring to ILs.^98^ Briefly, Brown and Brown mention that butylpyridinium cations are more symmetric than the [C_2_mim] cation and thus have a higher melting point. But how or why does symmetry impact melting points?

To quote Lopez and Yalkowsky:

“*…the strong relationship between symmetry and melting point reflects the fact that molecules are rotationally restricted in the crystal but not in the liquid. Since the symmetry number is equal to the ways that a molecule can be properly oriented for incorporation into the crystal lattice, it is a significant determinant of the melting point.*”^99^

To note here, is that symmetry can have different meanings (*e.g.*, point groups, space groups, rotational axes). Molecular descriptors based on existing symmetry elements are used to develop the theories discussed.^92,99,100^

In essence, Carnelley's Rule can be viewed as an early qualitative description of molecular packing efficiency: molecules with higher symmetry possess more equivalent orientations for lattice incorporation, thereby maximizing the probability of favorable close packing and minimizing configurational entropy in the solid state. From this perspective, the rule encapsulates a simple yet important structure–property relationship: symmetry facilitates crystallization.

In contrast, the deliberate introduction of asymmetry into IL cations, *via* the two alkyl chains, serves as a form of anti-Carnelley design, intentionally reducing symmetry to frustrate efficient packing and to increase the configurational and conformational entropy of the solid. This engineered disorder effectively lowers the lattice energy, leading to reduced melting points.^92^ Thus, cation asymmetry in ILs represents a deliberate anti-crystal-engineering strategy to reduce long-range order through a lack of molecular shape complementarity and to tune the balance between enthalpy and entropy. However, it should be noted that exceptions exist (*e.g.*, room-temperature ILs with highly symmetric ions) which demonstrate that low melting points can also arise from other design variables (*i.e.*, charge delocalization, weak H-bonding, conformationally flexible substituents), even when symmetry is increased.^101^ The degree of asymmetry, typically manifested through two unequal alkyl chains, introduces new modes of packing frustration and thermodynamic balance.

### 4.5 The valley of melting points

Purposefully introducing asymmetry illustrates how subtle variations in alkyl chain length reconfigure the balance between enthalpic stabilization and entropic disorder, a defining hallmark of molecular design in ILs. This interplay becomes evident through the emergence of chain-length-dependent patterns (*e.g.*, odd–even effects), where increasing chains lengths produces oscillations in melting behavior.

**Fig. 9:**
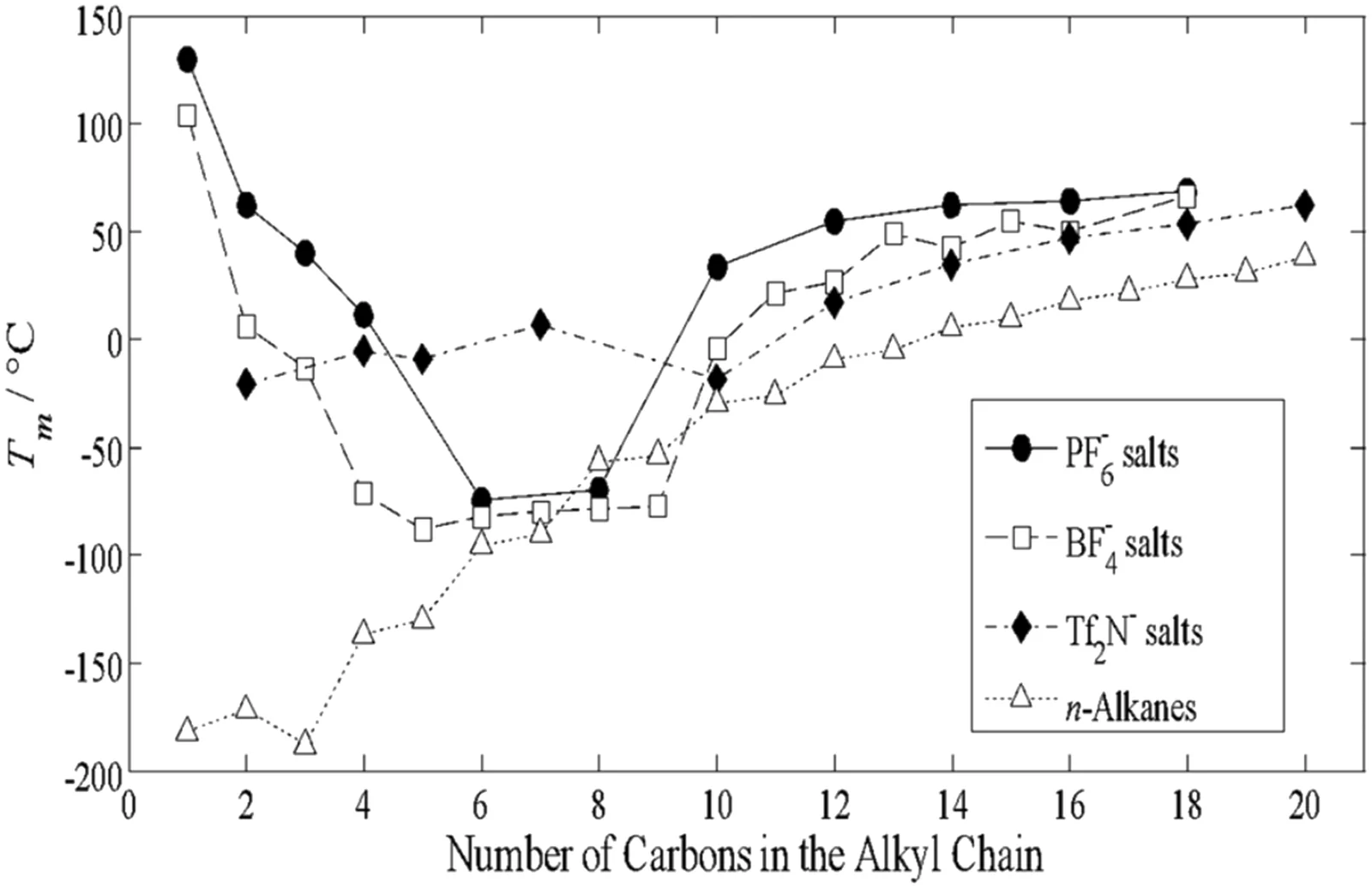
Melting point data for a dialyklated imidazolium ILs showing the influence of anion identity along with the discussed “alkyl-valley”. As alkyl chain length increases, a more predictable trend in melting points is observed. Reproduced from ref. 104 with permission from John Wiley and Sons, copyright 2010.

Building upon these principles, the melting points of di-alkylated imidazolium ILs bearing the [NTf_2_]^−^ anion (Fig. 9)^102,103^ reveal a “valley” trend: beginning with the butyl derivative, the melting point falls for the pentyl analog, rises again for the hexyl derivative, and continues in an alternating fashion.^104^ Once the chain length exceeds ten carbons, this pattern gives way to a more predictable progression, hinting at how chain length and molecular asymmetry jointly, yet subtly, dictate the crystallization landscape of ILs. More importantly, this also hints at how long-alkyl chains can be leveraged to promote crystallinity, pointing towards a fundamental crystal engineering variable (§ 5).

When the anion is changed from [NTf_2_]^−^ to more compact, symmetric species such as [PF_6_]^−^ or [BF_4_]^−^, the odd–even pattern effectively disappears and is replaced by a pronounced “low-melting valley”. Melting points drop sharply as the alkyl chain lengthens from C1 to C3, reach a minimum between C4 and C8, and then rise again as the chain extends beyond C10, ranges that are approximate rather than absolute. This valley reflects the shifting balance between enthalpic contributions from longer chains and entropic contributions from shorter chains^84^ with extended alkyl chains playing an important role in forming ordered solid-state structures.^79^ This alkyl-driven solidification of ILs is a key point of structural modification, using alkyl–alkyl interactions (*i.e.*, H⋯H contacts) to drive the crystallization of ILs. In essence we shift the balance of enthalpy and entropy by incorporating longer alkyl chains.

**Fig. 10:**
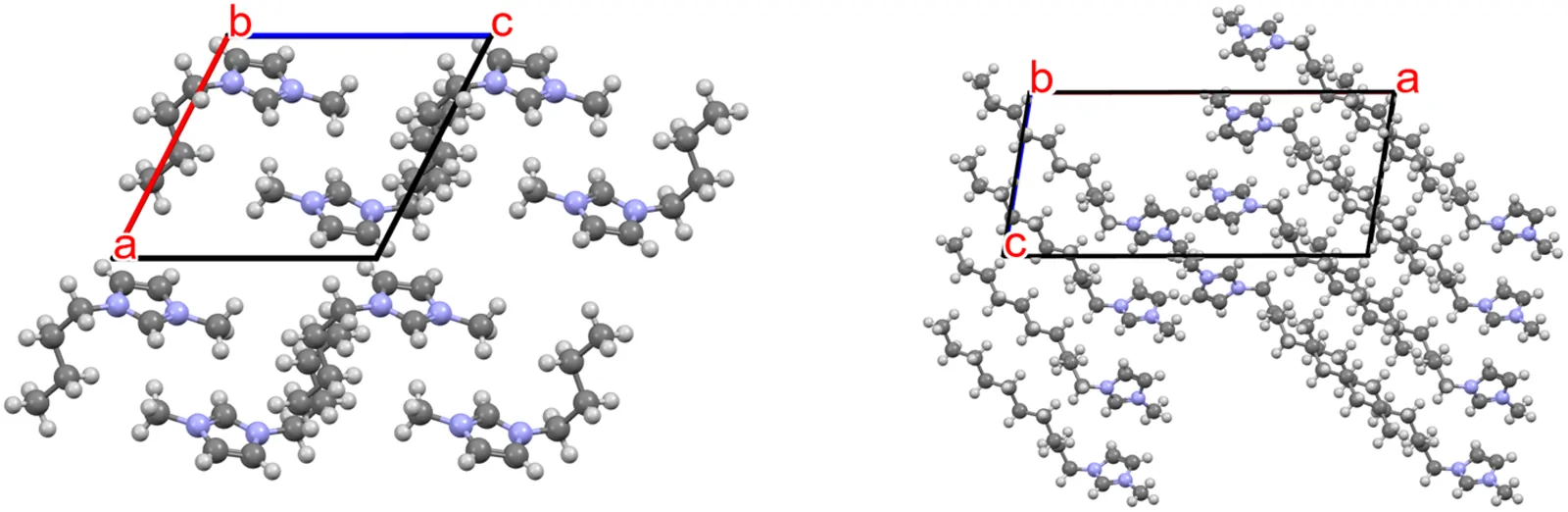
Packing diagrams of [C_4_mim] cations (from [C_4_mim][PF_6_], left)^106^*vs.* [C_10_mim] cations (from [C_10_mim][PF_6_], right).^42^ The influence of the alkyl chains on the formation of the crystalline solid is seen with the interdigitation in the [C_10_mim] packing diagram. Anions are omitted for clarity.

The influence of alkyl chains on the packing efficiency of ILs becomes clearer when a packing diagram of a [C_4_mim] derivative is compared with that of the [C_10_mim] analog (Fig. 10). While both structures show cation ordering, the interactions among alkyl chains are far more pronounced in the [C_10_mim] crystal. Nevertheless, inter-alkyl interactions are also present in [C_4_mim], both in the solid and liquid states.^105^ From the figure one can clearly see how the alkyl chains in [C_10_mim] align in an all-*trans* conformations, allowing for interdigitation and maximum overlap between the chains.

These observations highlight two key principles central to the crystal engineering of ILs. First, extended alkyl chains function as internal scaffolds, reinforcing the lattice through cooperative H⋯H and dispersive interactions that promote long-range order. Second, disrupting these interactions, whether by shortening the chains, introducing a chain kink (§ 5.2), or by adding functional groups (§ 5.3–5.6), frustrates the formation of these packing motifs. In doing so, such modifications deliberately weaken lattice cohesion and lower melting points, achieving the desired outcome of anti-crystal engineering: the controlled destabilization of crystalline order through molecular design.

The influence of long *versus* short alkyl chains further highlights the importance of conformation in dictating solid-state order. As the chains lengthen, their ability to adopt different torsional geometries—*gauche*, *eclipsed*, or *trans*—directly influences how efficiently cations can pack within the lattice. Put simply, these conformations either enable or frustrate the overlap of alkyl chains and the formation of crucial H⋯H contacts, interactions that play a defining role in stabilizing organic-based lattices.^53,107^ Within the language of crystal engineering, this highlights conformational control as a design strategy, one that allows fine-tuning of intermolecular overlap and packing efficiency to rationalize and allow, or frustrate, crystallization of ILs.
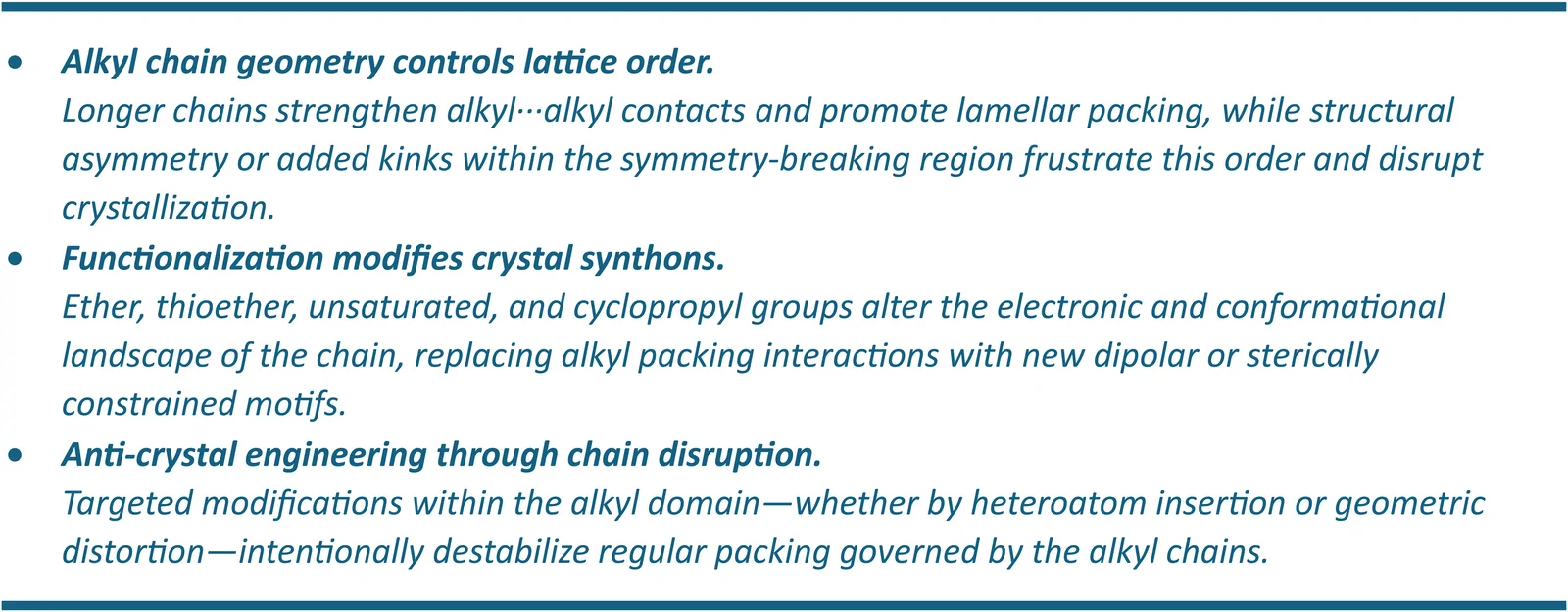


## 5 A long story short: crystal engineering with extended alkyl chains

As the anti-crystal engineering of ILs has continued to develop, several fundamental studies have examined the question of cation symmetry.^108,109^ The majority of these investigations focus on the molten state rather than solid-state structures. This is expected, as short-chain ILs have low melting points and are therefore challenging to examine in the crystalline state. Long-alkyl-chain ILs, however, are easier, though not easy by any means, to crystallize, and several key manuscripts have examined the solid-state properties of these compounds. Even when examined in the melt, the same shape anisotropy that disrupts efficient crystal packing underlies the observed low melting points*.*^110^

### 5.1 From lattice to liquid: structural continuity in ionic liquids

The correlation between solid-state packing and nanostructure in the molten phase is most apparent in ILs with long alkyl chains.^111^ These systems help rationalize the “alkyl valley” trend, where extended chains yield a more predictable progression of melting points. Scattering and simulation studies consistently reveal polar cationic networks interlaced with apolar alkyl domains that persist into the liquid phase, demonstrating that ILs are structured liquids.^56,105,112^ This segregation of domains is clearly evident when looking at packing diagrams of ILs (Fig. 10). The solid-state framework therefore provides a direct means to interpret the organization of the melt, showing how molecular geometry and conformational flexibility dictate both crystal formation and liquid order.

**Fig. 11:**
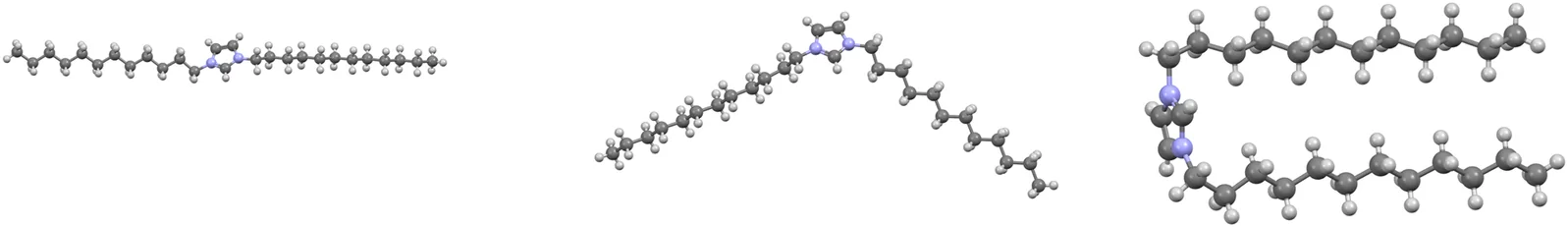
(left to right) Rod-, V-, U-shaped cation structures of symmetric dialkylated imidazolium cations.^114^ The anions (omitted for clarity) help form the distinct alkyl torsion angles, maximizing interaction between chains.

Mudring and co-workers offered key crystallographic insight by comparing asymmetric cations bearing methyl and dodecyl substituents with symmetric analogues containing two dodecyl chains.^113^ For these long chains, crystallization is driven by the maximization and ordering of H⋯H contacts among alkyl groups. Torsional changes along the chains produce distinct molecular shapes (rod-, V-, or U-shaped, Fig. 11) that determine how polar and nonpolar regions segregate in the lattice.^114^ U-shaped conformations restrict chain rotation through intramolecular contacts, reducing entropy and promoting ordered domains, whereas shortening one chain introduces asymmetry that increases entropy, weakens alkyl–alkyl packing, and lowers the melting point.

Symmetric long-chain cations therefore promote local self-ordering that propagates into long-range periodicity, a form of synthon templating wherein recurring alkyl⋯alkyl domains guide crystallization. The interplay between symmetry, chain length, and conformational anisotropy defines how subtle structural perturbations move ILs along the continuum between ordered solids and liquids.

### 5.2 The strongest kink in the chain

The discussion of the torsion angles leads into a larger discussion about the impact of the chains on crystallinity. As noted earlier, the cation of an IL can be broadly divided into three structural domains: the charge-rich aromatic core, the symmetry-breaking region, and the hydrophobic region. Herein we focus the discussion, and impact, of the symmetry-breaking and hydrophobic regions.

The hydrophobic-region draws back to the alkyl-valley concept discussed earlier. Once the side-chain length reaches roughly eight to ten carbons, alkyl–alkyl (*viz.*, H⋯H) contacts begin to drive the packing of ILs.^115^ The impact of the hydrophobic region, from a crystal engineering perspective, is complex. As the chain grows, dispersion forces strengthen while coulombic forces remain essentially constant; beyond a critical alkyl length, the balance shifts progressively, though never entirely, away from electrostatic control.^116^ In essence, the alkyl–alkyl interactions begin to energetically become more controlling. Further, the longer chains also increase the charge–charge separation and change the cation's symmetry, key components affecting physicochemical properties.^111^

The symmetry-breaking domain of the alkyl chain comprises the first two to eight atoms between the charge-dense headgroup and the hydrophobic tail. Structural modifications introduced here (branching, unsaturation, heteroatom substitution, cyclopropyl rings; see below) reduce effective molecular symmetry and add conformational freedom. These features frustrate close packing, weaken cohesive van der Waals contacts near the headgroup, lower lattice enthalpy (Δ*H*_fus_), and increase the entropy of fusion (Δ*S*_fus_), collectively destabilizing the crystal.^117^

The central concept for this section is that disruption of the energetically preferred *anti* conformation of the alkyl chains exerts a profound effect on the crystallinity of ILs. In their lowest-energy state, long alkyl chains adopt an extended zig-zag geometry that maximizes van der Waals H⋯H contacts and promotes cohesive alkyl–alkyl aggregation. Functional group substitution that perturbs this conformation introduces conformations that destabilize chain interactions and impede efficient packing. Such disruptions weaken the enthalpic driving force for lattice formation and increase configurational entropy, collectively disfavoring crystallization. Viewed through the lens of anti-crystal engineering, perturbing the *anti* conformation of the alkyl chain is a targeted act of disorder, an intentional means to frustrate packing and suppress the very interactions that drive crystallization.

### 5.3 Ether and thioether groups

Incorporating heteroatoms such as oxygen or sulfur into the alkyl side chains of imidazolium ILs was initially envisioned as an anti-crystal-engineering strategy: localized polarity and lone-pair repulsion would frustrate the lamellar alkyl packing that stabilizes crystalline phases. Crystallographic and thermophysical studies show the outcome is more complex and depends strongly on substitution position, alkyl chain length, and secondary interactions.

**Fig. 12:**
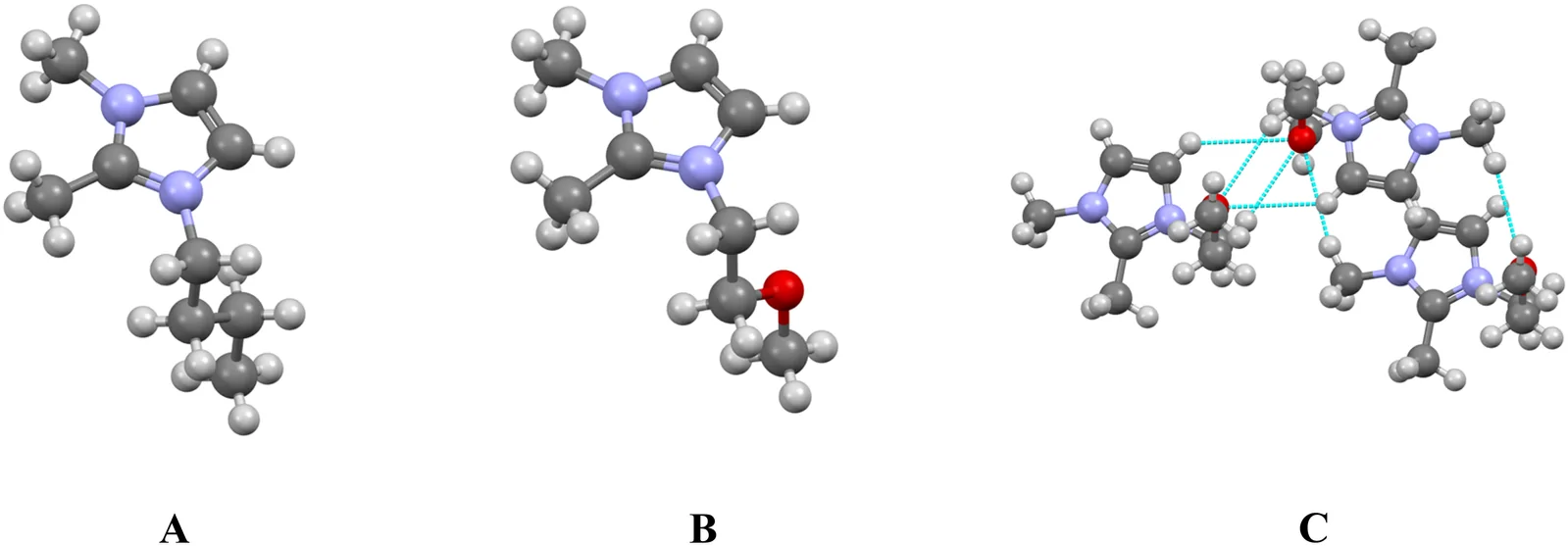
(A) The [C_4_dime] cation; (B) the [C_4_dime] cation with an ether moiety at the 3 position of the alkyl chain; (C) representation of the H⋯O interactions formed from the ether moieties in the alkyl chains. All structures used in this image were reported by Henderson *et al.*^118^

In early work on [C_4_dime][Cl], insertion of an ether moiety at the 3-position of the alkyl chain resulted in melting points increasing from 373 K to 430 K.^118^ Repulsive O⋯O contacts reorganized the chains, enabling compensatory C–H⋯O interactions between ether oxygens and the methyl and methylene sites (Fig. 12). A subsequent report by Fei *et al.*^119^ later observed similar behavior in polyether-functionalized salts, where H⋯O hydrogen bonds between cations increased lattice cohesion. The effect was noted to depend on anion identity. Introduction of oxygen also modifies the cation dipole moment and electrostatic potential,^120^ slightly biases the C–O–C linkage toward the *gauche* geometry (≈0.5 kcal mol^−1^ over *anti*), and thereby inserts a structural “kink” in the symmetry-breaking domain of the alkyl chain that can, in theory, disrupt packing. Of note, there do exist examples where ether functionalize ILs exhibit lower melting points, as expected following the anti-crystal engineering principles discussed.^121^

Sulfur substitution exerts analogous but more pronounced effects. Thioethers lower melting points most effectively when the S-atom resides within the symmetry-breaking domain close to the headgroup.^122^ Because C–S–C prefers the *gauche* orientation even more strongly than C–O–C,^123^ sulfur introduces more conformational kinks and greater packing frustration. At the same time, sulfur can engage in weak H⋯S hydrogen bonds^124^ and σ-hole (chalcogen-bond) interactions^125,126^ with neighboring anions, blending dispersive and electrostatic contacts. These competing forces—repulsion, polarization, and new interaction motifs—define the delicate thermodynamic balance governing crystallinity.

**Fig. 13:**
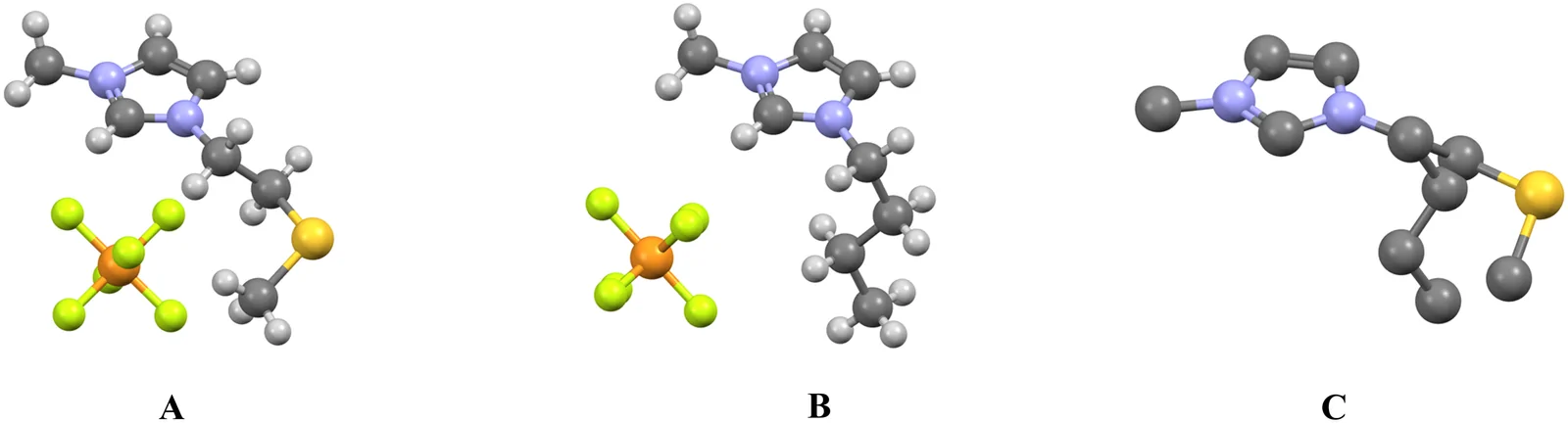
Depiction of the impact of thioether groups on the alkyl chains of ILs. (A) The [C_4_mim][PF_6_] derivative with a sulfur at the C3 position of the alkyl chain^131^ (B) the [C_4_mim][PF_6_] structure^106^ (C) overlay of the two cations to emphasize the change in torsion angles on the chain.

A total of nine structures are reported in the CSD wherein a thioether moiety is incorporated into the alkyl chain of an imidazolium IL. Three of the reports deal with the synthesis and characterization of ILs,^127–129^ two are from carbene related studies wherein imidazolium cations are precursors,^130,131^ and the final is simply a structure deposited in the CSD.^132^ Notably, all of the reported structures have the thioether moiety within three carbons of the imidazolium cation, well within the symmetry breaking region. As noted earlier, the position of the substituent strongly modulates its ability to induce liquefaction: thioethers proximal to the cation core exert a smaller melting-point depression than those located further along the chain. Even so, the [C_4_mim][PF_6_] derivative bearing a C3-thioether substituent adopts the expected *gauche* conformation, directly evidencing the conformational bias and hinting at the packing frustration introduced by sulfur substitution (Fig. 13).

### 5.4 Unsaturation (alkenes)

Introducing a *cis* double bond into a long alkyl chain reliably decreases melting points, a tactic borrowed directly from naturally occurring unsaturated fatty acids. For instance, *cis*-oleic acid melts near 13 °C, whereas its *trans* isomer elaidic acid melts near 45 °C and the saturated analogue stearic acid near 69 °C. The marked depression arises because a *cis* C=C bond introduces a rigid kink that disrupts the regular zig-zag alignment and prevents tight alkyl stacking.^133^ An analogous effect occurs in IL.

del Río *et al.* compared imidazolium salts bearing oleyl (*cis*) and stearyl (saturated) chains and observed substantially lower melting points for the *cis* derivatives.^134^ Although crystallization of such long-chain ILs is difficult, single crystals of several saturated analogues showed lamellar packing dominated by alkyl–alkyl interactions, establishing the structural baseline for chain-driven lattice organization.

Alkene position along the chain also provides a means of tuning melting behavior.^117^ Mirjafari *et al.* found that placing a *cis* double bond at C9 of an octadecyl chain produced a room-temperature liquid (*T*_m_ ≈ −21 °C).^135^ Placement of a *cis* alkene at the C9 position of the alkyl chain resulted in a significant reduction of melting points, resulting in a room temperature IL (*T*_m_ ≈ −21 °C). This site lies at the boundary of the symmetry-breaking region, where a kink most effectively disrupts cooperative alkyl interactions, effectively extending the symmetry breaking region and shortening the hydrophobic domain. To note, *trans*-alkenes produce a smaller but still significant melting-point reduction.^117,136^ The effect likely reflects a combination of increased rigidity, altered dipole moment, shorter effective chain length, and reduced interdigitation.^137,138^

**Fig. 14:**
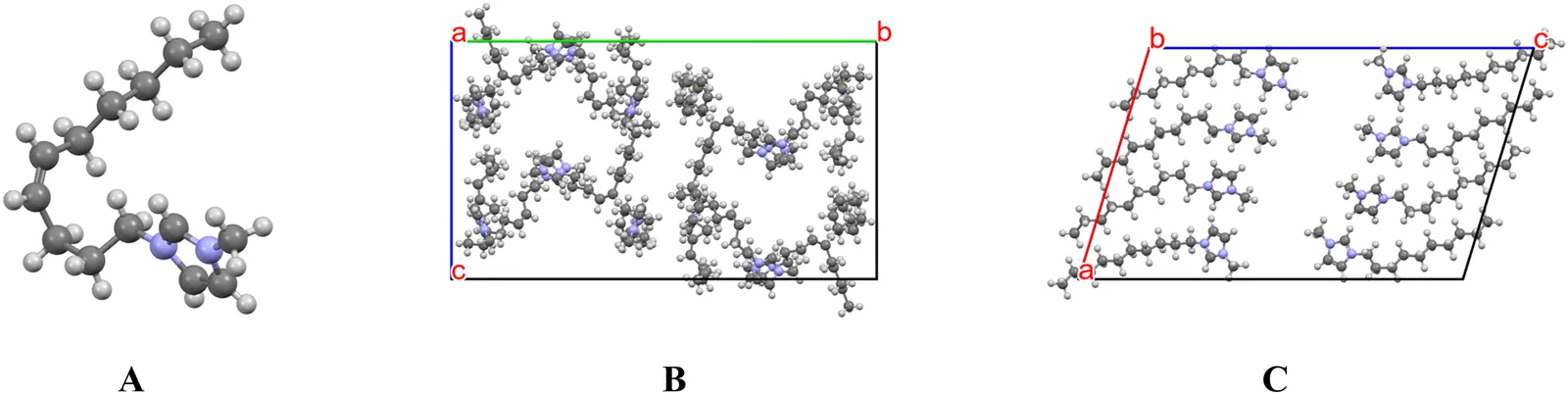
(A) The cation of a dialkylated IL with a *cis*-alkene in the chain.^104^ (B) Packing diagram of a *cis*-alkene bearing IL viewed down the crystallographic *a* axis contrasted with the fully saturated derivative (C) viewed down the *B* axis. Anions removed for clarity. The influences of the kinked alkyl chain is evident when contrasting the packing patterns of the two compounds. Disruption of alkyl interactions *via* chain kinking lowers melting points.

The only reported single-crystal structure of a *cis*-alkene-bearing IL, from Jim Davis's group, features a C4 *cis* double bond within a decyl chain on an imidazolium [BPh_4_] salt. The packing diagram for this compound clearly shows the chain bend responsible for reduced crystallinity (Fig. 14).^104,117^ Contrasting the saturated and unsaturated derivatives points towards the strong influence these longer alkyl chains have on the formation of the crystalline state of ILs.

Incorporating a double bond into an alkyl chain, especially in the *cis* configuration, is one of the most reliable ways to reduce crystallinity in ILs. The rigid kink created by the C=C bond breaks the regular zig-zag packing of alkyl chains, disrupting the strong alkyl⋯alkyl interactions that normally stabilize the solid. The position of the double bond is also important: kinks around C8–C10 cause the greatest disruption and yield the lowest melting points. In crystal-engineering terms, unsaturation acts as an *anti-crystal-engineering* tool—it removes a stabilizing packing pattern and replaces it with controlled molecular disorder, lowering lattice stability and promoting liquid formation.

### 5.5 Cyclopropanation

Introducing C=C bonds within bis-alkylated ILs lowers melting points but also increases susceptibility to oxidation. Nature provides a parallel solution: through homeoviscous adaptation, certain bacteria preserve membrane fluidity by converting unsaturated lipids into cyclopropanated analogues that retain chain kinks.^139^ Cyclopropane rings impose comparable conformational disruptions on alkyl chains, interrupting linear packing and reducing lattice stability.

**Fig. 15:**
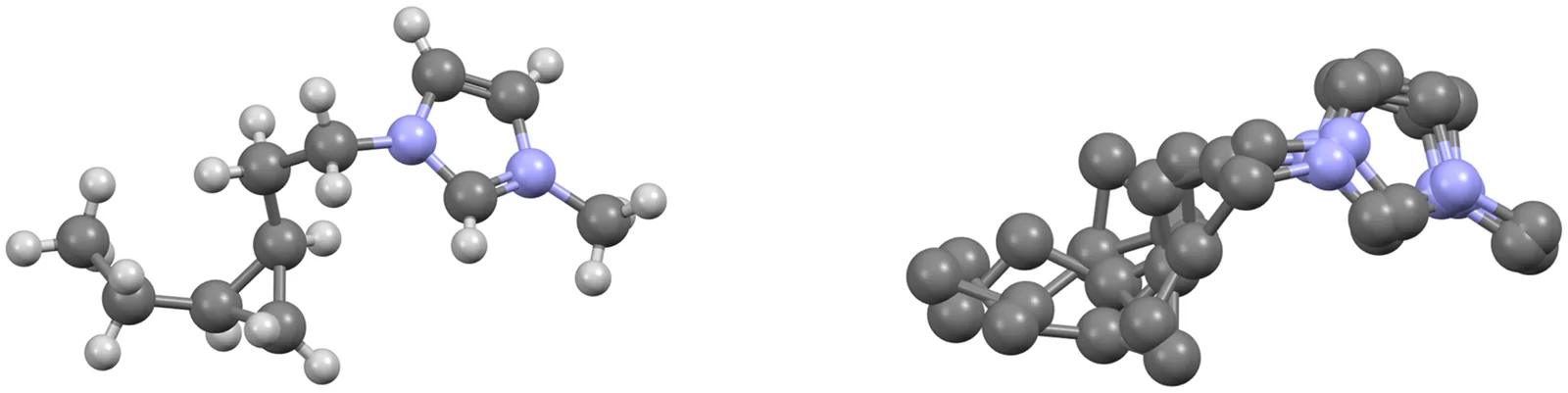
(left) The cation of an imidazolium-based IL bearing a cyclopropanated alkyl chain. A cyclopropane ring forces a kink in the alkyl chain frustrating alkyl chain packing and thus lowering melting points. (right) The disordered cation moiety showing multiple alkyl conformations. Reproduced from ref. 141 with permission from American Chemical Society, copyright 2023.

There are very few single-crystal structures of a dialkylated imidazolium IL containing a cyclopropane ring, though they are becoming more prevalent.^140^ One example has a hexyl side chain bears a cyclopropane moiety between the C4 and C5 positions.^141^ The alkyl chain is highly disordered, adopting several conformations around the ring (Fig. 15). As in the *cis*-alkene case, the cyclopropane introduces a rigid kink that frustrates alkyl-chain alignment and depresses the melting point. Although typical H⋯H contacts remain, the constrained torsion angles generate sufficient steric distortion to override these stabilizing interactions and hinder crystallization.

From a crystal-engineering standpoint, cyclopropanation represents a rigid-bond anti-crystal-engineering motif. It preserves the symmetry disruption and packing frustration associated with unsaturation while improving chemical robustness. Because the substitution is purely hydrocarbon, it minimally perturbs dipole moment and avoids new polar synthons, isolating the geometric origin of the crystallization barrier. Cyclopropane substitution thus provides a straightforward, chemically stable route to tune lattice disorder and disrupt crystallization.

## 6 Reflections and curiosities in crystal engineering of imidazolium cations

In the course of writing this review, several observations and ideas emerged that, while tangential to the main narrative, highlight the broader relevance of crystal-engineering principles.

### 6.1 The magic methyl effect

The dramatic influence of methylation, for instance, is not unique to ionic liquids. Schönherr and Cernak have discussed the “magic methyl effect” in pharmaceuticals, where adding a single methyl group dramatically alters half-life, solubility, and bioavailability.^142^ A similar pattern appears in ILs: targeted methylation of imidazolium cations can modify thermal stability, crystallinity, and solubility, while methylation along long alkyl chains often lowers melting points.^140^ Thus, the methyl group, chemically simple yet structurally potent, acts as a fine-tuning element for symmetry, sterics, and packing in both biological and ionic systems.

Curiously, derivatives methylated at the C4/5 positions of the imidazolium ring remain scarcely explored, despite being known natural products.^143^ Synthetic cost and complexity likely explain this gap, though it underscores how much untapped ground remains in IL design. Meaningful comparisons, however, require deliberate structural control to isolate the effects of methylation from other substituent changes.

### 6.2 On the odd–even effect and experimental convention

We specifically discussed the odd–even effect with respect to alkyl chains, and the subtle, yet noticeable influence it has on the physicochemical properties of ILs. In this light, we feel it worthwhile sharing the fantastic report by Profs Gautam Desiraju and Jack Dunitz. In their paper, they examine the broader scope of the odd–even effect with respect to total number of carbons in a molecule, not just in an alkyl chain.^144^ In the words of Desiraju and Dunitz:

“*…the even/odd disparity is unlikely to rest on any fundamental parity rule but seems to result mainly from the activities of the organic chemists who have succeeded in synthesizing such a vast number of compounds. The even ones are on the whole easier to prepare than the odd ones.*”

Maybe the IL community's preference for using even chains is simply because they are easier (or cheaper) to make and we all collectively choose the path of least resistance. Maybe it is convention or tradition to use even alkyl chains. Maybe we, as humans, just prefer even numbers.^145^ In any case, given the impact of the odd–even effect, we would encourage future studies to consider adding in odd alkyl chains, leveraging the small yet important structural impacts they may impart.

### 6.3 Polymorphism, supercooling, and reproducibility

Given the prevalence of polymorphism in ILs, careful documentation of crystallization methods and reproducibility is essential. As McCrone observed, the number of polymorphs discovered is proportional to the time spent searching for them, while Bernstein provides the important reminder about ‘disappearing polymorphs’.^146,147^

Thermal history and sample preparation play critical roles in these outcomes.^148–150^ For instance, the literature reports over sixty melting points for [C_4_mim][NTf_2_], ranging from −9 to 78 °C, an astonishing spread that reflects differences in sample purity, thermal treatment, polymorphic form, though also intentional mixtures to raise melting points. However, consistent reporting of crystallization methods and standardized characterization protocols would greatly enhance the reproducibility and comparability of IL data.

Supercooling further complicates interpretation. Many ILs, owing to high viscosity and slow molecular dynamics, bypass nucleation and persist as supercooled liquids for months or longer.^151^ Such kinetic trapping can blur the distinction between solid and liquid phases, leading to cases where a nominal “liquid” is a metastable solid that never crystallized. Supercooling thus epitomizes the balance between molecular mobility and packing order, reminding us that the apparent liquid state may often reflect kinetic barriers rather than fundamental disorder.^152,153^

### 6.4 Intermolecular forces and the role of the anti-crystal engineer

Melting points are a balance between solid-state interactions (Δ*H*_fus_) and the entropy gained on melting (Δ*S*_fus_). Crystal packing and intermolecular forces strongly influence both terms, thus crystal structures can provide key details for ILs^154^ With the appropriate caveats in mind one can readily glean important information about interactions in IL crystal structures and link them to observed properties. One important point that bears attention is the discussion of intermolecular forces and the temptation to use them to over-simplify melting points (or other properties).^155^ It is within this energetic window that the *anti-crystal engineer* operates—strategically disrupting or redirecting weak interactions to tune lattice stability.

While a hydrogen bond may contribute tens of kJ mol^−1^, coulombic attractions account for hundreds or even thousands.^156^ Non-covalent interactions comprise perhaps 20–30% of the total lattice energy,^27,28^ but within that modest window lies the playground of the anti-crystal engineer.^157,158^ Disrupting a subset of these weaker interactions lowers melting points; conversely, introducing specific directional contacts can either depress or raise them. The challenge, of course, is disentangling the effects of subtle structural changes from the complex interplay of electrostatics and dispersion that defines ILs.

## 7 At the end, the beginning

The work of Paul Walden, while debated,^159^ is often cited as the genesis of the field of ILs with his study of ethylammonium nitrate as a room temperature IL.^160^ Indeed many of the cited works herein have discussed the impact of this manuscript on the field of ILs as an origin. With the principles learned, hopefully, from the present manuscript, this salt is perhaps a mysterious finding due to its low melting point. Upon cursory inspection, this room temperature IL does not appear to make sense given our modern structural understanding of the formation of ILs. It is a relatively small molecule with practically no sterics, it does not bear any charge disperse cation (like imidazolium), it has short linear alkyl groups, and is paired with a small non-bulky anion. Yet, it has a melting point below room temperature (*T*_m_ ≈ 12 °C). Given the prominence of this salt on the field of ILs, it is perhaps not surprising that a crystal structure of the salt was grown and analyzed.^161^

Examining the crystal of ethylammonium nitrate, and comparing it with other related structures, a theory as to the melting process for the compound was formed. In brief, Henderson *et al.* theorized that due to initially limited motion of the cation, the H-bonds between the ions are weakened with increasing thermal energy, which then leads to increased volume followed by rotations beginning in the methyl and ammonium head-group. This motion, in turn, disrupts the lattice resulting in a molten state. This melting mechanism was proposed using multiple crystal structures gathered at increased temperatures, in conjunction with Raman spectroscopy.

The crystallographic study of ethylammonium nitrate points towards an important idea: crystallography is more than a way to obtain atomic coordinates and connectivity. Through detailed analysis, especially when combined with complementary methods, a robust understanding of structure–property relationships can emerge. In this case, a framework for elucidating the melting ‘mechanism’ is revealed, pointing to the underlying thermodynamic features that govern the process.

## 8 Conclusion and outlook

This review has examined the structural principles that shape ionic-liquid behavior: rotational freedom of alkyl chains, hydrogen-bond formation, alkyl packing disruptions, cation methylation, the entropic effects of small chains, electronic structure, and cation asymmetry. These ideas represent the core heuristics for rational IL design, that is anti-crystal engineering design. Crystallography supports this goal by providing exact structural information and enabling quantitative links between molecular architecture and bulk properties.

Advances in diffraction instrumentation and computation have ushered in a renaissance of structure-based IL design. Detailed analysis of crystal packing,^85^ intermolecular interactions,^162^ and structural motifs^163^ continues to clarify the principles of anti-crystal engineering—how weakly coordinated ions and conformationally flexible cations yield low-melting yet functional materials.

Looking ahead, crystallography will remain central to IL research, particularly as the field converges with data science. Machine-learning approaches require large, well-annotated datasets, and solved crystal structures provide exactly that – rich geometric descriptors such as bond lengths, torsion angles, asphericity, globularity, and void space. A broad set of free tools (for example: CrystalExplorer,^164^ Crystal Grower,^165^ MiCMoS^166^) enables detailed analysis of intermolecular forces in crystals, yielding additional features for training sets. As algorithms and prediction software advance, structure generation and screening will likely become routine, allowing pre-synthesis evaluation of lattice stability and packing tendencies. Gavezzotti's demonstration of an entirely synthetic crystallographic dataset^167^ hints at this future of simulation-driven exploration.

In the coming years, structure-based design will integrate fully into the discovery and optimization of task-specific ILs. The combination of automated crystallographic analysis, high-throughput synthesis, and rational molecular design will accelerate progress from structural hypothesis to verified material. The continued dialogue between crystal engineering and ionic-liquid chemistry promises not only better understanding of existing systems but also the guided creation of new ones—where order, disorder, and function are deliberately balanced from the outset.

## Conflicts of interest

There are no conflicts to declare.

## Data Availability

The data used within this manuscript is freely available through the Cambridge Structural database. No new data has been presented.
